# Sex-dependent regulation of cholinergic responses in the mouse colon: functional evidence for reduced acetylcholinesterase-dependent control and nitrergic modulation in females

**DOI:** 10.1007/s10974-026-09739-w

**Published:** 2026-09-18

**Authors:** Stherfanni Almeida Lima-Moura, José Eduardo da Silva-Santos

**Affiliations:** Laboratory of Cardiovascular and Smooth Muscle Biology, Department of Pharmacology, Universidade Federal de Santa Catarina – Campus Universitário Trindade – Center of Biological Sciences – Block D, Room 116, Rua João Pio Duarte Silva, 241, Florianópolis, Santa Catarina 88037-000 Brazil

**Keywords:** Cholinergic signaling, Nitric oxide, Enteric nervous system, Dysmotility, Sex differences, Colonic smooth muscle

## Abstract

Despite the higher incidence of diseases affecting the digestive tract among women, sex-related differences in gastrointestinal motility remain poorly investigated. This study compared the contractile and relaxant effects of cholinergic, adrenergic, and dopaminergic stimuli across major gastrointestinal segments of mice. For this, female and male Swiss mice (3–4 months old) were anesthetized and euthanized for isolation of gastric corpus strips (~ 0.5 cm length) and segments (~ 1.5 cm length) of the jejunum, ileum, and colon (*n* ≥ 6/group), which were mounted in organ baths under resting tension of 0.5 g. Spontaneous activity and responses to cumulative or noncumulative concentrations (100 pM–300 µM) of acetylcholine (ACh), carbamylcholine (CCh), norepinephrine (NOR), isoprenaline (ISO), and dopamine (DA) were recorded. Additionally, responses to electrical field stimulation (EFS; 20 V, 1 ms, 1–128 Hz, 10 s) were assessed in colonic segments before and after incubation with 1 µM neostigmine or 100 µM L-NAME. Although only slight sex-associated differences were found for the effects of NOR, ISO, DA, or ACh in the gastric corpus, jejunum, and ileum, the colon of female mice was hyperresponsive to ACh, but not to CCh. Furthermore, compared with male preparations, colonic segments from female mice displayed enhanced contractile responses and reduced relaxation following EFS. EFS-induced contractions were more sensitive to neostigmine and L-NAME in preparations from male mice. In conclusion, reduced acetylcholinesterase and nitrergic activity contribute to the enhanced cholinergic responses in the female colon, providing mechanistic insight into sex differences in gastrointestinal motility.

**Supplementary Information:**

The online version contains supplementary material available at https://doi.org/10.1007/s10974-026-09739-w.

## Introduction

Gastrointestinal motility results from the coordinated interplay between smooth muscle cells, interstitial cells of Cajal, and the neurons that comprise the enteric nervous system. Locally produced neurotransmitters, hormones, immune mediators, and microbial metabolites directly or indirectly modulate these cell populations, ultimately shaping the balance between contractile and relaxing responses and, consequently, the intensity and frequency of gastrointestinal movements (for reviews, see Margolis et al. 2021; Sanders et al. 2014; Spencer and Hu 2020).

Dysmotility refers to abnormal gastrointestinal motor activity that is not attributable to mechanical obstruction. Several disorders present hypermotility as a prominent feature, and the list of diseases associated with hypomotility is even broader (for review see Azpiroz and Malagelada 2016; Lazcano-Ocampo et al. 2026; Xu et al. 2024). Additionally, severe systemic conditions such as advanced stages of chronic kidney disease and sepsis are marked by profound gastrointestinal hypomotility. Dysmotility disrupts normal propulsion, mixing, and emptying of intestinal contents, significantly affecting intestinal transit and nutrient absorption.

According to epidemiological data, the prevalence of gastrointestinal diseases is generally higher in women than in men. These conditions include, but are not limited to, irritable bowel syndrome (Oka et al. 2020), gastroparesis (Parkman et al. 2019), chronic constipation (Zakari et al. 2017), and esophageal reflux (Nasseri-Moghaddam et al. 2008), all of which are directly associated with impaired gastrointestinal motility. Interestingly, under physiological conditions, women exhibit slower gastric emptying and intestinal transit compared with men (Graff et al. 2001). This sex difference seems to depend on hormonal status, as it is absent in postmenopausal women (Hutson et al. 1989; Sadik et al. 2003). The reasons underlying these sex-related differences, however, remain poorly understood, largely because experimental studies have historically omitted female animals. However, the high density of estrogen receptors in gastric smooth muscle cells in females appears to account for enhanced activation of the nitric oxide-guanylate cyclase pathway, which can delay intestinal motility (Al-Shboul et al. 2018).

Although the role of female sex hormones, estrogen receptors, and the nitric oxide pathway in gastrointestinal motility has been previously reported (Li et al. 2016), no functional studies to date have directly compared the reactivity of male and female gastrointestinal segments to most of the major neurotransmitters involved in the physiological regulation of gut motility. In this study, we explored the hypothesis that cholinergic, adrenergic, and dopaminergic systems differ in their contractile and relaxant effects on the gastrointestinal system of male and female mice. Our findings reinforce marked sex-related and region-specific differences in the physiological regulation of gastrointestinal contractility.

## Methods

### Animals, experimental groups, and ethical approval

We used 3–4-month-old female (35–55 g) and male (40–60 g) *Swiss* (*Mus musculus*) mice supplied by the animal vivarium of Universidade Federal de Santa Catarina (Florianópolis, SC, Brazil). The animals were housed in acrylic ventilated cages (Alesco Indústria e Comércio Ltda, Monte Mor, SP, Brazil), subjected to a 12-h light-dark cycle, and monitored temperature (21 ± 1 °C), with unrestricted access to chow and water until the day of the experiments. We conducted all experimental blocks using gastrointestinal segments from randomly selected male and female mice. Our Institutional Animal Care and Use Committee authorized all experimental procedures described here (CEUA/UFSC, authorization number 5366250520). We used 136 mice (68 female and 68 male) for this study. This number does not include animals used during preliminary experiments to establish and optimize the experimental model and protocols.

### Drugs and physiological nutritive solutions

L-(−)-norepinephrine (+)-bitartrate salt monohydrate (NOR), isoprenaline hydrochloride (ISO), dopamine hydrochloride (DA), acetylcholine chloride (ACh), carbamylcholine hydrochloride (CCh), neostigmine (NEO), and all salts used to prepare the nutritive solutions were obtained from Sigma-Aldrich (Saint Louis, MO, USA). The regular physiological salt solution (PSS, pH 7.4) used in our experiments contained (in mM): NaCl 131.3, KCl 4.7, KH_2_PO_4_ 1.18, MgSO_4_·7H_2_O 1.17, NaHCO_3_ 14.9, Dextrose 5.5, CaCl_2_·2H_2_O 1.6, and EDTA 0.0088. We also used a 120 mM KCl-modified solution, in which KCl and NaCl concentrations were adjusted to 119.9 and 14.4 mM, respectively. All stock solutions of drugs were prepared according to the manufacturer’s instructions and diluted in PSS before use in our experiments.

### Removal of the gastrointestinal tract and setup for functional evaluation

**Fig. 1:**
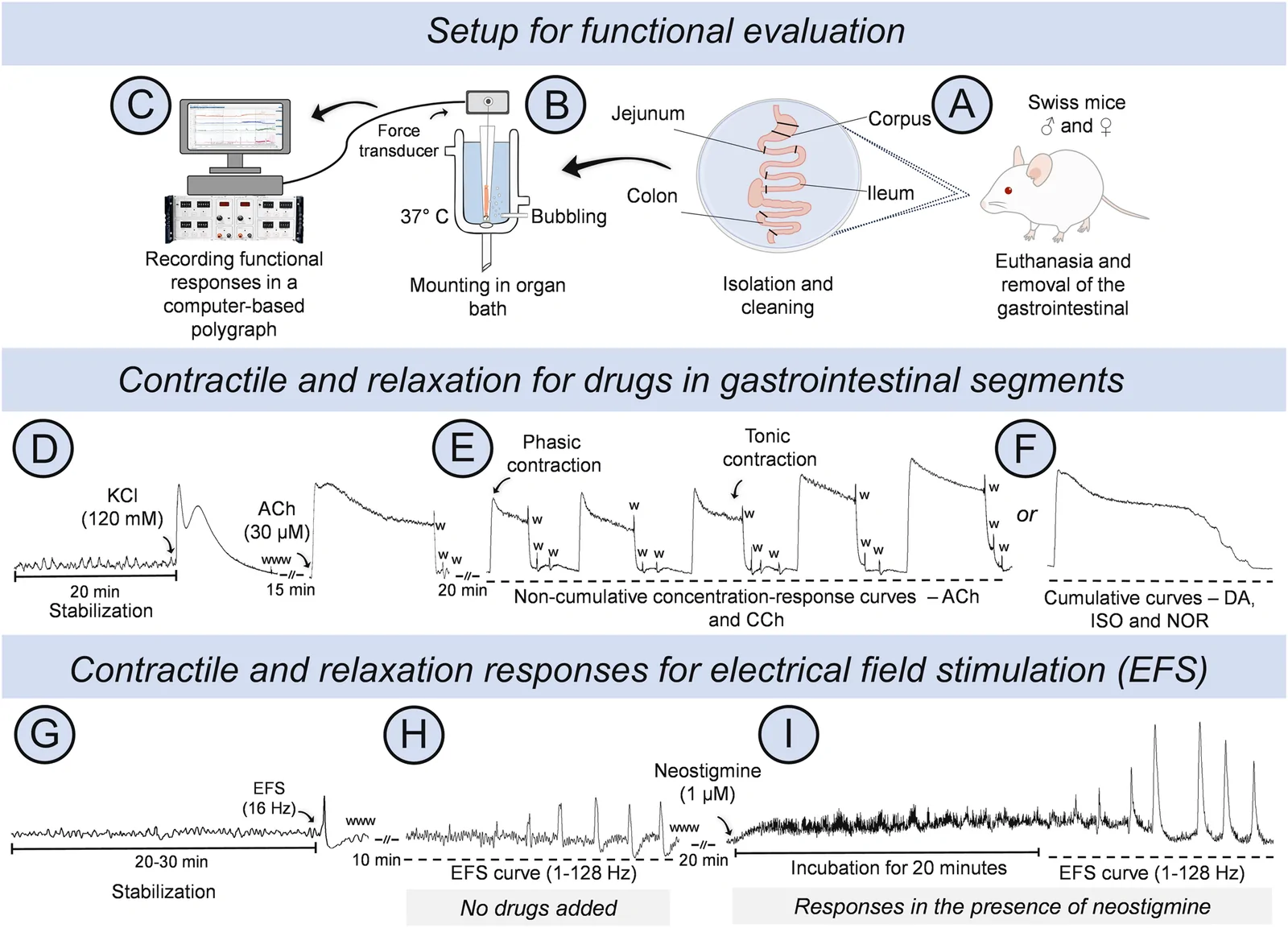
Schematic illustration of the experimental protocols used to evaluate gastrointestinal reactivity. Male and female mice were euthanized by anesthesia overdose followed by cervical dislocation, and the stomach, jejunum, ileum, and colon were collected. The gastrointestinal segments were separated, cleaned, and placed in a Petri dish containing physiological saline solution (PSS) at 37 °C (**A**). Tissue segments measuring 2–5 mm (stomach) or 15–20 mm (jejunum, ileum, and colon) were mounted in organ bath chambers **(B**) filled with PSS, continuously bubbled with atmospheric air, maintained at 37 °C, and equilibrated under a resting tension of 0.5 g. Functional responses were recorded in a computer-based polygraph (**C**). For drug-stimulation experiments, tissues were allowed to stabilize for 20 min, then exposed to PSS containing 120 mM KCl, followed by 30 µM acetylcholine (ACh) to confirm functional viability (**D**). Contractile responses (**E**) to noncumulative concentrations (1 nM–300 µM) of ACh or carbamylcholine (CCh), and relaxing responses (**F**) to cumulative concentrations (1 nM–300 µM) of dopamine (DA), isoprenaline (ISO), or norepinephrine (NOR), were evaluated in separate preparations. In experiments using electrical field stimulation, a 20–30 min stabilization period was allowed, and the viability of colonic segments was assessed by their response to a single 16 Hz stimulus (**G**). This was followed by the construction of frequency–response curves obtained before (**H**) and during incubation with neostigmine (**I**). Each “w” inserted above the traces indicates the washing step, during which the bath solution was replaced with fresh PSS

To obtain the organs used in functional studies, the mice received a single intraperitoneal injection containing 5 mg ketamine and 1 mg xylazine. Once the animals were anesthetized, euthanasia was performed by cervical dislocation. We then immediately assessed the abdominal cavity and excised the gastrointestinal tract from the stomach to the colon, placing it in a beaker containing warm (~ 37 °C) PSS. The organ was washed and put in a Petri dish for removal of connective tissues and fat, and the stomach, jejunum, ileum, and colon were dissected (Fig. 1A). The gastric and intestinal lumen were gently flushed with PSS, and the organs were reaccommodated in beakers containing fresh PSS. The stomach was sectioned to isolate the corpus, which was cut into ~ 2 mm wide by ~ 5 mm long strips. The jejunum, ileum, and colon were sectioned into 15–20 mm long segments. We used a surgical needle and cotton thread to carefully fix a small loop on one side and an approximately 15 cm-long line at the other end of the organs. The cotton thread loop was used to secure the preparations on the longitudinal axis in organ baths containing 4 mL of PSS, maintained at 37 °C, and constantly bubbled with an aquarium air pump (Fig. 1B). The line at the other end of the organ was connected to force transducers (model MLT0201, Panlab, Barcelona, Spain). The transducers were coupled to an amplifier and a computerized recording system (Fig. 1C) along with their application program (models ML228, PowerLab8/30, and LabChart^®^ Pro v. 7.3.3, respectively), all from AD Instruments (Castle Hill, Australia). Prepared in this way, the system continuously records the spontaneous movements (expressed as waves/min) and tone (expressed in grams) of the preparations, allowing the quantification of both contractile and relaxation responses evoked by either drug (as shown by typical traces in Fig. 1D-F) or electrical field stimulation (as shown in typical traces in Fig. 1G–I). All preparations were adjusted for a resting tension of 0.5 g when accommodated in the organ bath and allowed to stabilize for 20–30 min (Fig. 1D and G) before any experimental intervention. After the experimental protocols, the segments were removed from the baths, cleaned with disposable paper towels, and weighed. The wet weight of each preparation was used for individual normalization of contractile responses, as described ahead. In experiments using pharmacological stimuli, each preparation received only one drug, either a contractile or relaxing agent.

### Measurement of the spontaneous activity

**Fig. 2:**
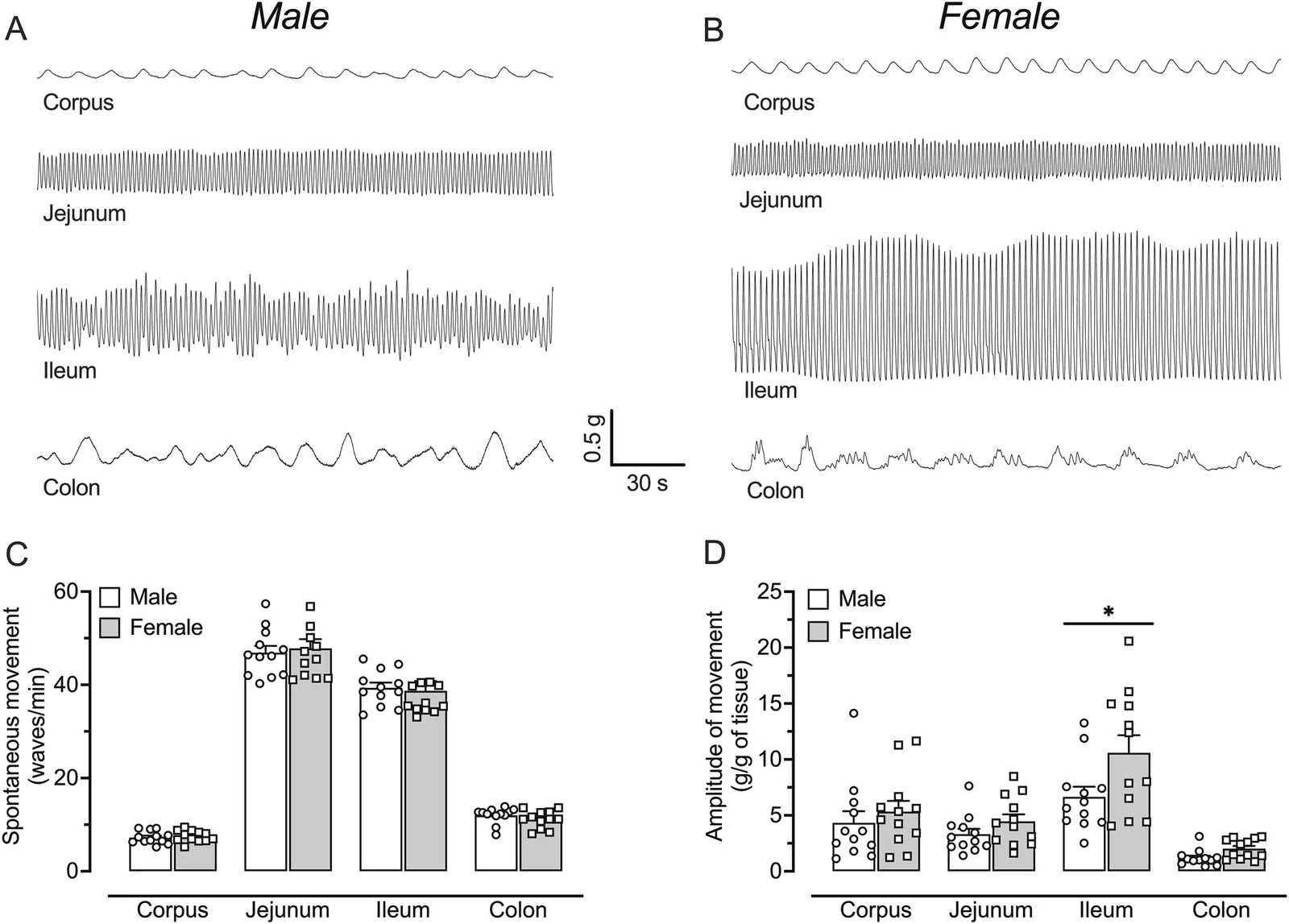
Spontaneous activity of gastrointestinal preparations. Representative trace recordings showing the waves formed by the spontaneous contractile activity of stomach, jejunum, ileum, and colon segments from male (**A**) and female (**B**) mice. The frequency (**C**) and amplitude (**D**) of spontaneous movements were quantified and compared across gastrointestinal segments and between sexes. Data show the mean values recorded over 10 min. The amplitude of spontaneous movements was normalized to the tissue weight of each preparation. Results are presented as mean ± standard error of the mean (SEM) from tissues obtained from 12 animals per group. * indicates *P* < 0.05; two-way ANOVA followed by Šidák’s post hoc test

The basal activity profile was evaluated during the stabilization period in the organ baths (Fig. 1D) before stimulation with any drug. For gastrointestinal preparations, the muscle movement is well defined as waves continuously generated by the spontaneous contractile and relaxation activity (see Fig. 2A and B for original trace recordings). The average frequency of these waves was evaluated over 10 min and expressed as spontaneous movements (in waves/min). We also measured wave amplitude, quantified as the force developed (in g), normalized to the weight of each organ, and expressed as g of contraction/g of tissue.

### Determination of contractile responses to cholinergic drugs

**Fig. 3:**
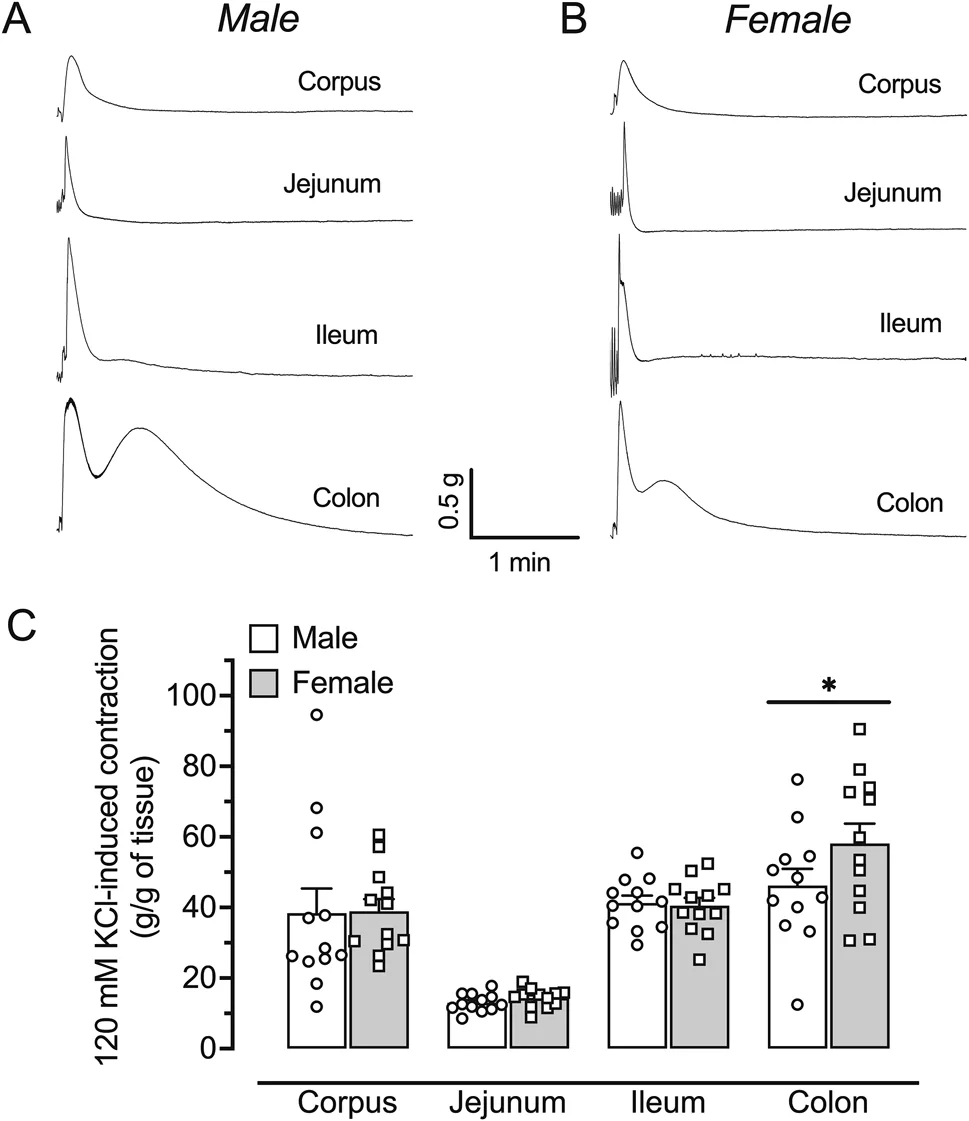
Contraction induced by PSS containing 120 mM KCl in gastrointestinal preparations of male and female mice. Representative trace recordings illustrating depolarization-induced contraction by KCl in each gastrointestinal segment (**A** and **B**), and maximal KCl-induced responses recorded in preparations from male and female (**C**) mice. Data are presented as the mean ± standard error of the mean, along with dot plots, obtained from tissues of 12 animals per group. * indicates *P* < 0.05; two-way ANOVA followed by Šidák’s post hoc test

After 20 min of stabilization, all preparations subjected to pharmacological approaches were initially exposed to a modified nutritive solution containing 120 mM KCl (see Fig. 3A and B for original trace recordings). Once the tissues reached maximal contractile response, the preparations were washed with PSS and, 15 min later, stimulated with 30 µM ACh for 5 min. The initial contractile responses to KCl and ACh were used to confirm tissue viability (Fig. 1D). Then, we washed the baths again and allowed a new 20-min resting period. Afterward, the preparations were sequentially exposed to ACh or CCh at concentrations of 0.01, 0.03, 0.1, 0.3, 1, 3, 10, 30, 100, and 300 µM. Each concentration was kept in the bath for sufficient time to allow development of both the peak (phasic) and the sustained (tonic) contraction, as indicated in Fig. 1E. The preparations were washed three times with PSS and allowed to return to the basal tone before we added the next concentration into the bath. This approach was used to construct noncumulative concentration-response curves for these cholinergic agonists. Both phasic and tonic responses were quantified and expressed as the contraction normalized by the weight of each organ (g/g of tissue).

### Evaluation of the relaxation responses to adrenergic and dopaminergic drugs

After stabilization, the tissues received the same initial stimulus with the modified high KCl nutritive solution and ACh, as detailed above (Fig. 1D). After this, the preparations were washed three times with PSS and allowed to rest for an additional 15-minute interval. Then, the preparations were stimulated again with 30 µM ACh and, during the resulting tonic contraction, we added cumulative concentrations of norepinephrine, isoprenaline (both from 100 pM to 30 µM) or dopamine (10 nM to 3 mM) into the baths (Fig. 1F). Relaxation responses were expressed as a percentage, normalized to the maximal tonic contraction elicited by ACh.

### Electrical field stimulation studies

We used only colon segments in these experiments. The organs were prepared and mounted in organ baths as previously described and positioned between bipolar electrodes connected to an electrical stimulator (LE 12406, Panlab, Barcelona, Spain) to enable electrical field stimulation (EFS). In this study, EFS was set to a constant amplitude of 20 V, 1-ms pulse width, trains of stimuli lasting 10 s, and a variable (1–128 Hz) number of pulses per second. After stabilization, the colon segments received a single 16 Hz stimulation train to confirm tissue viability (Fig. 1G). After a new 10-minute resting period, the preparations were subjected to sequential pulse trains at 1, 2, 4, 8, 16, 32, 64, and 128 Hz, which elicited contractile and relaxation responses. Importantly, each subsequent electrical pulse was generated only after muscle tone returned to basal values, allowing recording of frequency-dependent response curves (Fig. 1H). After the first set of EFS, the preparations were washed with fresh PSS and incubated with vehicle (PSS only) or 1 µM neostigmine for 20 min, when the frequency-dependent response curve was obtained again (Fig. 1I). A similar set of experiments was performed in preparations incubated with 100 µM L-NAME for 20 min. This approach allowed the investigation of both contractile and relaxing responses mediated by myenteric nerves in response to EFS. Both the peak of the relaxation and the peak of the contraction evoked by the electrical pulses were measured, normalized by the weight of each organ, and expressed as g of contraction/g of tissue. We also quantified the length and the area under the curve of each response (see Supplementary Fig. S1 for details).

### Statistical analysis

All data included in this study were obtained from tissues of at least six different animals per group. Results showing contractile and relaxation responses are presented as the mean ± standard error of the mean (SEM). Whenever possible, the data were also displayed as dot plots. The half-maximal effective concentration (EC_50_) was obtained by interpolation of data from either noncumulative or cumulative concentration-response curves, and is expressed in µM, together with the 95% confidence interval, or the mean ± SEM of the negative logarithm of the EC_50_ (pD2). Supplementary material includes tables containing detailed descriptive statistics and additional comparisons. Results were analyzed using two-way analysis of variance (ANOVA), followed by Šídák’s post hoc test or Student’s *t*-test, as appropriate. A *p-*value less than 0.05 was considered statistically significant. The graphs were generated and the statistical analyses were performed using GraphPad Prism version 10.0 (GraphPad Software, Boston, MA, USA). Figure legends provide details on the sample size of each experimental group and the statistical tests used.

## Results

### Regional analysis and sex-related differences of spontaneous gastrointestinal movements

In the first series of analyses, we evaluated the frequency and magnitude of spontaneous movements in gastrointestinal segments from male and female mice, as shown in the representative recordings in Fig. 2A and B. Notably, the frequency of spontaneous contractions was higher in the jejunum and ileum than in the gastric corpus and colon, with no sex-related difference within any segment (Fig. 2C). Assessment of spontaneous contraction amplitude, a direct measure of force generated, revealed no differences between males and females in the gastric corpus, jejunum, or colon (Fig. 2D). In contrast, the amplitude was significantly greater in the ileum of females (Fig. 2D). Detailed descriptive statistics and additional comparisons are provided in Supplementary Table S1.

### Comparison of the effects of KCl on the gastrointestinal smooth muscle tone

KCl exposure resulted in a monophasic contractile response in the gastric corpus, jejunum, and ileum, and a biphasic contraction in the colon segments from both male and female mice, illustrated by the typical recordings included in Fig. 3A and B. Regional comparisons showed that jejunal reactivity to KCl was lower than that observed in other gastrointestinal segments, with a similar pattern in preparations from male and female mice (Fig. 3C; see Supplementary Table S2 for descriptive statistics and detailed comparisons). Although there were no sex-related differences in the maximal contractile responses reached in the gastric corpus, jejunum and ileum, KCl-induced contractions were significantly greater in the colon of female mice than in males (Fig. 3C).

### Comparison of the effects of muscarinic agonists on the gastrointestinal smooth muscle tone

Incubation of the gastrointestinal tissues with ACh resulted in well-defined contractile responses characterized by an initial (phasic) and a sustained (tonic) component. The profiles of these two phases did not differ between corresponding segments from male and female mice, as shown by the time course of responses to 30 µM ACh in Supplementary Fig. S2.

**Fig. 4:**
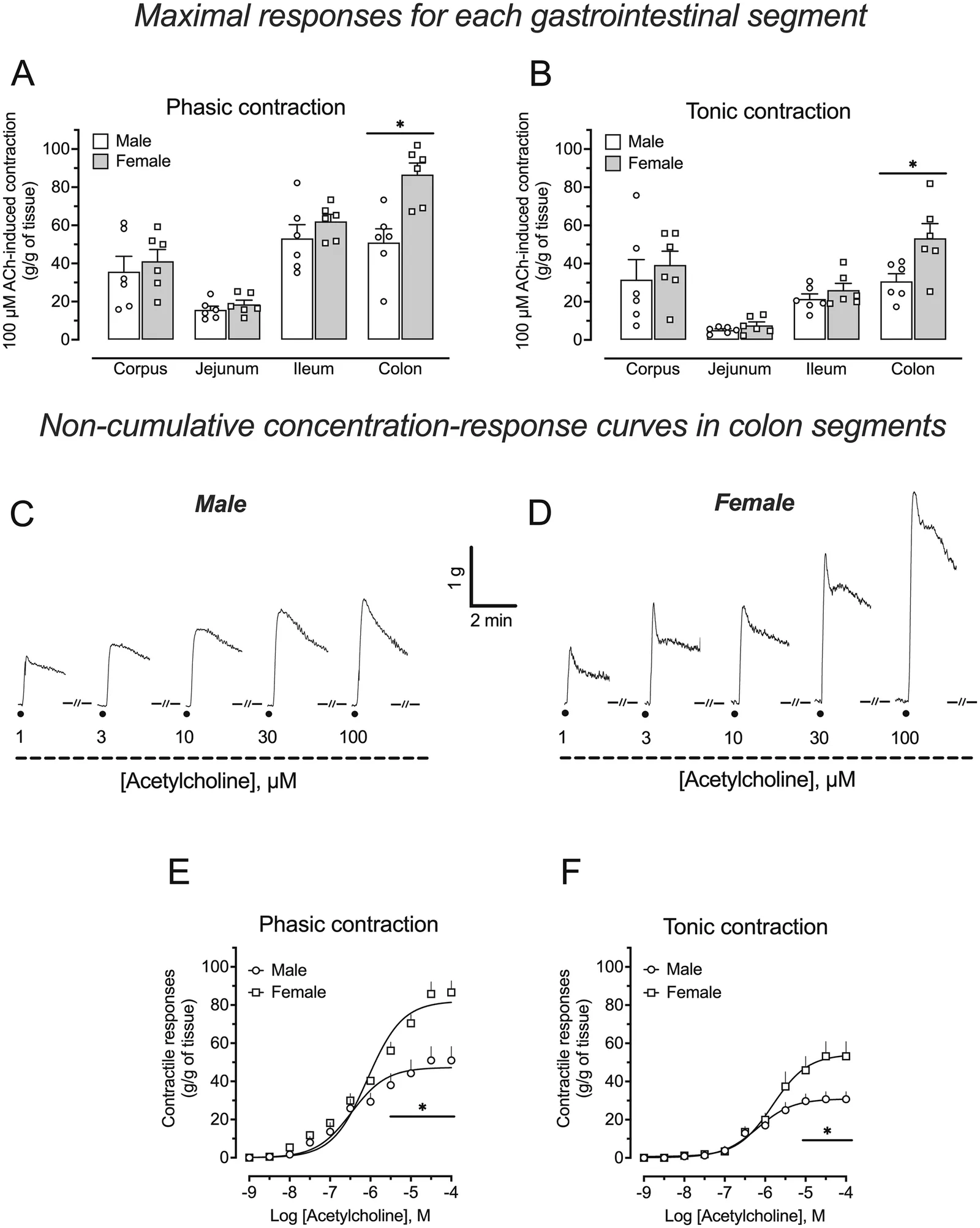
Contractile effects induced by acetylcholine in gastrointestinal segments of male and female mice. Maximal phasic (**A**) and tonic (**B**) contractions evoked by acetylcholine (ACh) in gastric corpus, jejunum, ileum, and colon preparations of male and female mice. Representative trace recordings illustrating the noncumulative concentration-response curve to ACh in colonic segments of male (**C**) and female (**D**) mice. Comparison of the phasic (**E**) and tonic (**F**) components of the noncumulative concentration-response curve to ACh (1 nM–300 µM) in colonic preparations from male and female mice. Data are presented as the mean ± standard error of the mean, with dot plots shown in panels A and B. Six animals were used per group. * indicates *P* < 0.05; two-way ANOVA followed by Šidák’s post hoc test

The magnitude of the maximal phasic (Fig. 4A) and tonic (Fig. 4B) contractile responses to ACh varied among gastrointestinal segments, with the lowest responses observed in the jejunum. Interestingly, although the maximal phasic and tonic contractile effects induced by ACh did not differ between males and females in the gastric corpus, jejunum, or ileum, both responses were enhanced in the colon of females compared with that of males (Fig. 4A and B). Trace recordings, showing the noncumulative concentration-response curve for ACh-induced contractions in the colon of male (Fig. 4C) and female (Fig. 4D) mice, revealed that this augmented reactivity was not apparent at the lowest concentrations but became evident at concentrations ranging from 3 to 100 µM ACh, as quantified in Fig. 4E and F. Indeed, the Emax for both phasic and tonic components of ACh-induced contraction was approximately 50% greater in the colon of female mice. Supplementary Tables S3 and S4 provide the descriptive statistics of the noncumulative concentration-response curves obtained for all gastrointestinal segments included in this study.

**Fig. 5:**
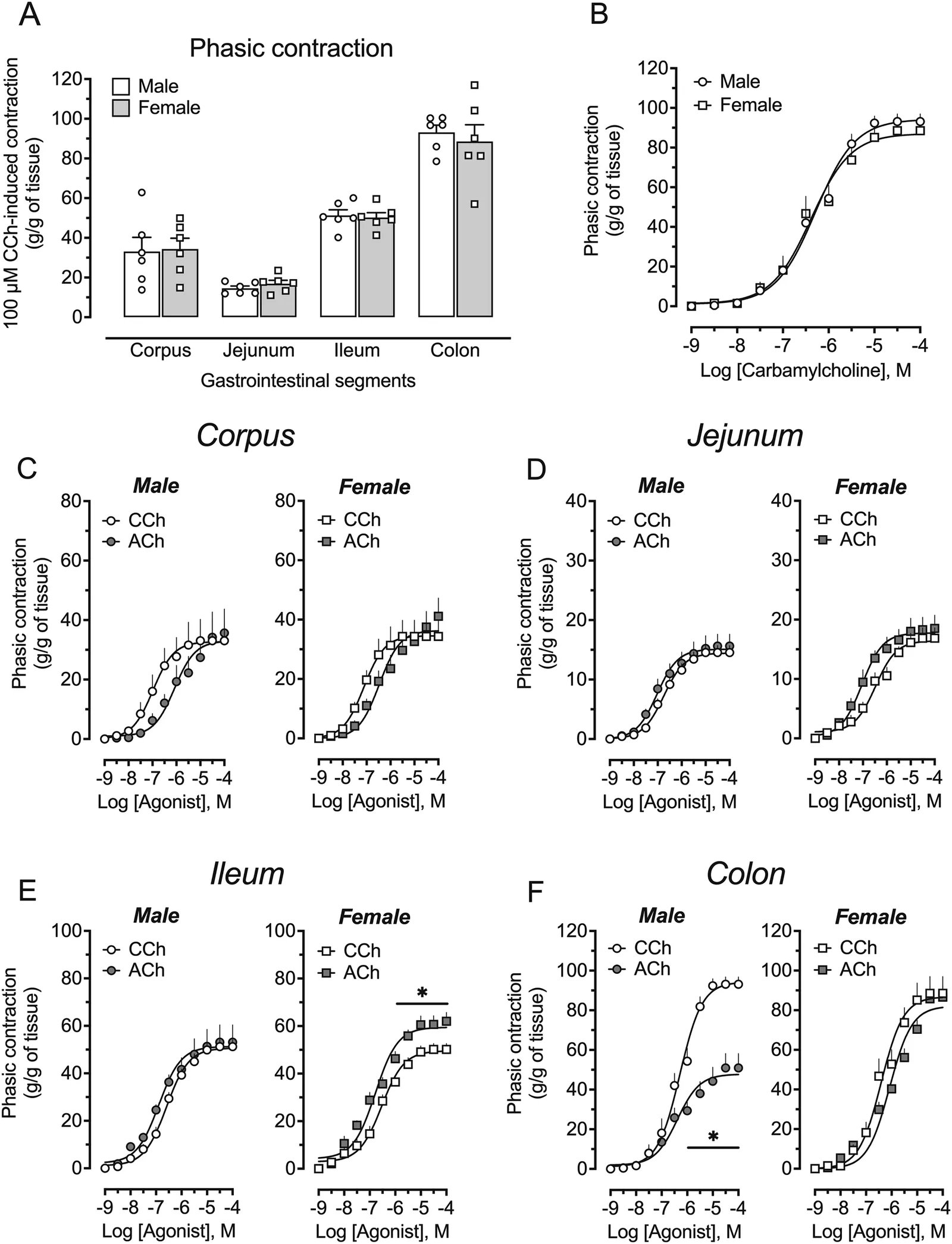
Contractile effects induced by carbamylcholine in gastrointestinal segments of male and female mice. Maximal contractile responses to carbamylcholine (CCh) in gastric corpus, jejunum, ileum, and colon preparations from male and female mice (**A**), and noncumulative concentration-response curves obtained in colonic preparations (**B**). For direct comparison, contractile responses evoked by noncumulative concentrations of CCh and acetylcholine (ACh) in gastric corpus (**C**), jejunum (**D**), ileum (**E**), and colon (**F**) from male and female mice are plotted together. All data included in this figure refer to the phasic component of the contractile responses. Results are presented as the mean ± standard error of the mean, with dot plots shown in panel A. Intestinal segments from 6 animals were used per group. * indicates *P* < 0.05; two-way ANOVA followed by Šidák’s post hoc test

CCh, another nonselective muscarinic agonist, also evoked contractile responses, resembling the pattern observed for ACh, with the lowest reactivity found in the jejunum for both the phasic (Fig. 5A) and tonic contractions (Supplementary Fig. S3A). Nevertheless, noncumulative concentration-response curves to CCh revealed no differences in the contractile responses obtained of colonic segments from male and female mice (Fig. 5B). A closer inspection of these data revealed that, while no sex-related differences were observed in the contractile effects of equimolar concentrations of ACh and CCh in the gastric corpus (Fig. 5C), jejunum (Fig. 5D), and ileum (Fig. 5E) segments, the colon of male – but not of female – mice exhibited greater contractile responses to CCh than to ACh (Fig. 5F). A similar profile was also found for the tonic contractions (Supplementary Fig. S3B-C). Descriptive statistics and additional comparisons of the maximal effects and the EC_50_ values for ACh and CCh are included in Supplementary Tables S5 and S6. These analyses indicate that, although CCh and ACh differ in efficacy, their potencies are similar in the colon of male mice.

### Comparison of relaxation induced by dopaminergic and adrenergic ligands in gastrointestinal segments

**Fig. 6:**
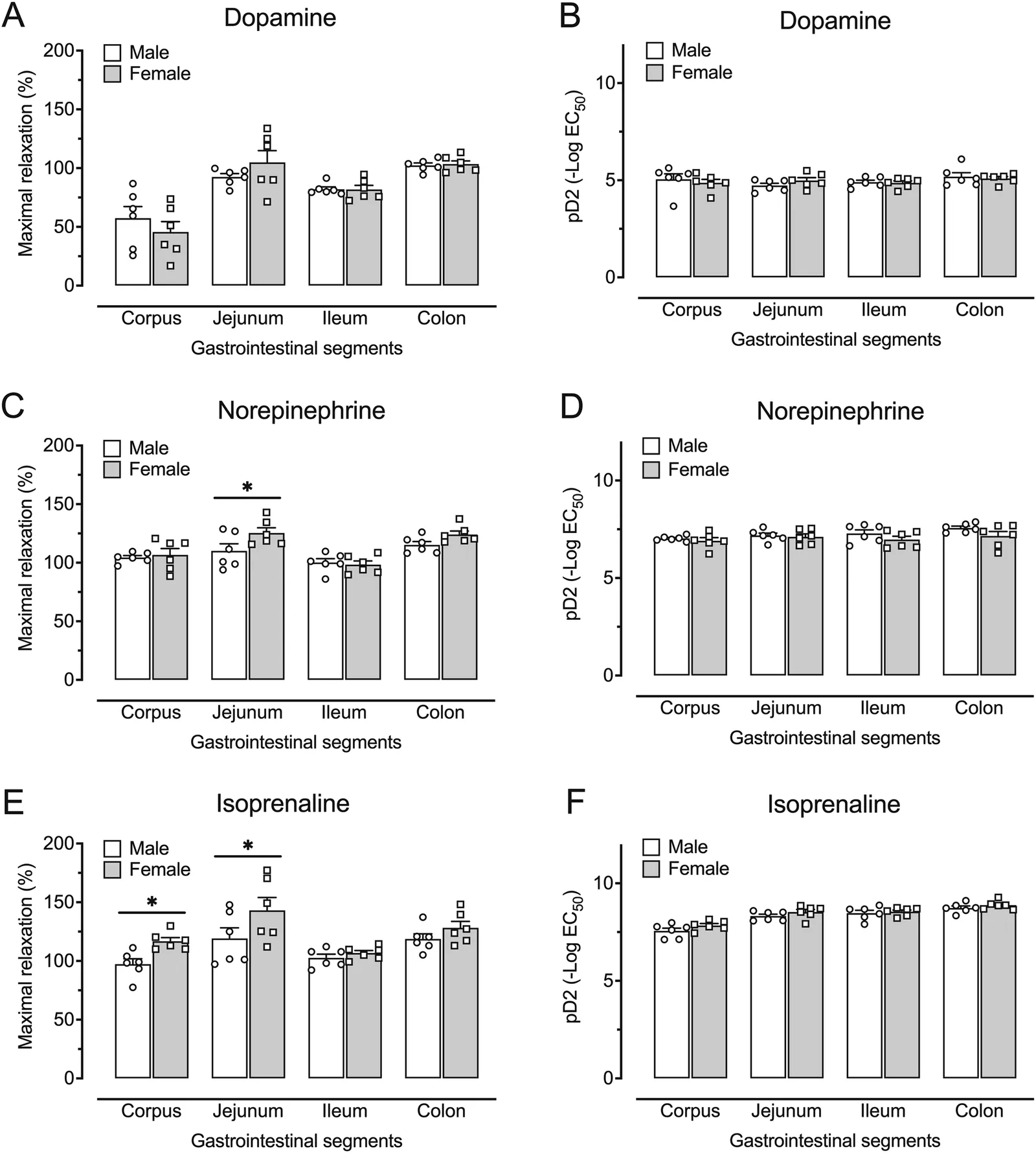
Relaxant responses obtained in gastrointestinal segments from male and female mice. Maximal relaxation responses and corresponding pD2 values obtained in response to dopamine (**A**, **B**), norepinephrine (**C**, **D**), and isoprenaline (**E**, **F**) in gastrointestinal segments from male and female mice. All data included in this Figure were derived from cumulative concentration-response curves constructed during the tonic contraction evoked by 30 µM acetylcholine. Results are presented as the mean ± standard error of the mean, with individual data points shown as dot plots. Intestinal segments from 6 animals were used per group. * indicates *P* < 0.05; two-way ANOVA followed by Šidák’s post hoc test

Dopamine-induced relaxation was less effective in the gastric corpus (Emax ~ 50%) than in intestinal segments (Emax ~ 80–100%), with no sex-related differences (Fig. 6A). On the other hand, both norepinephrine (Fig. 6C) and isoprenaline (Fig. 6E) induced full relaxation in all gastrointestinal preparations evaluated. Comparisons of the Emax values for norepinephrine in the jejunum (Fig. 6C) and for isoprenaline in the gastric corpus and jejunum (Fig. 6E) revealed greater maximal relaxation responses in tissues from female mice.

Although our data showed that dopamine was approximately 1,000-fold less potent than the adrenergic ligands, and that isoprenaline was more potent than norepinephrine in nearly all gastrointestinal segments (see Supplementary Table S7 for detailed comparisons), no sex-related differences were detected in the potency of any of the agonists between preparations from male and female mice (Fig. 6B, D and F)

### Sex-related differences in EFS-induced contractile and relaxing responses

**Fig. 7:**
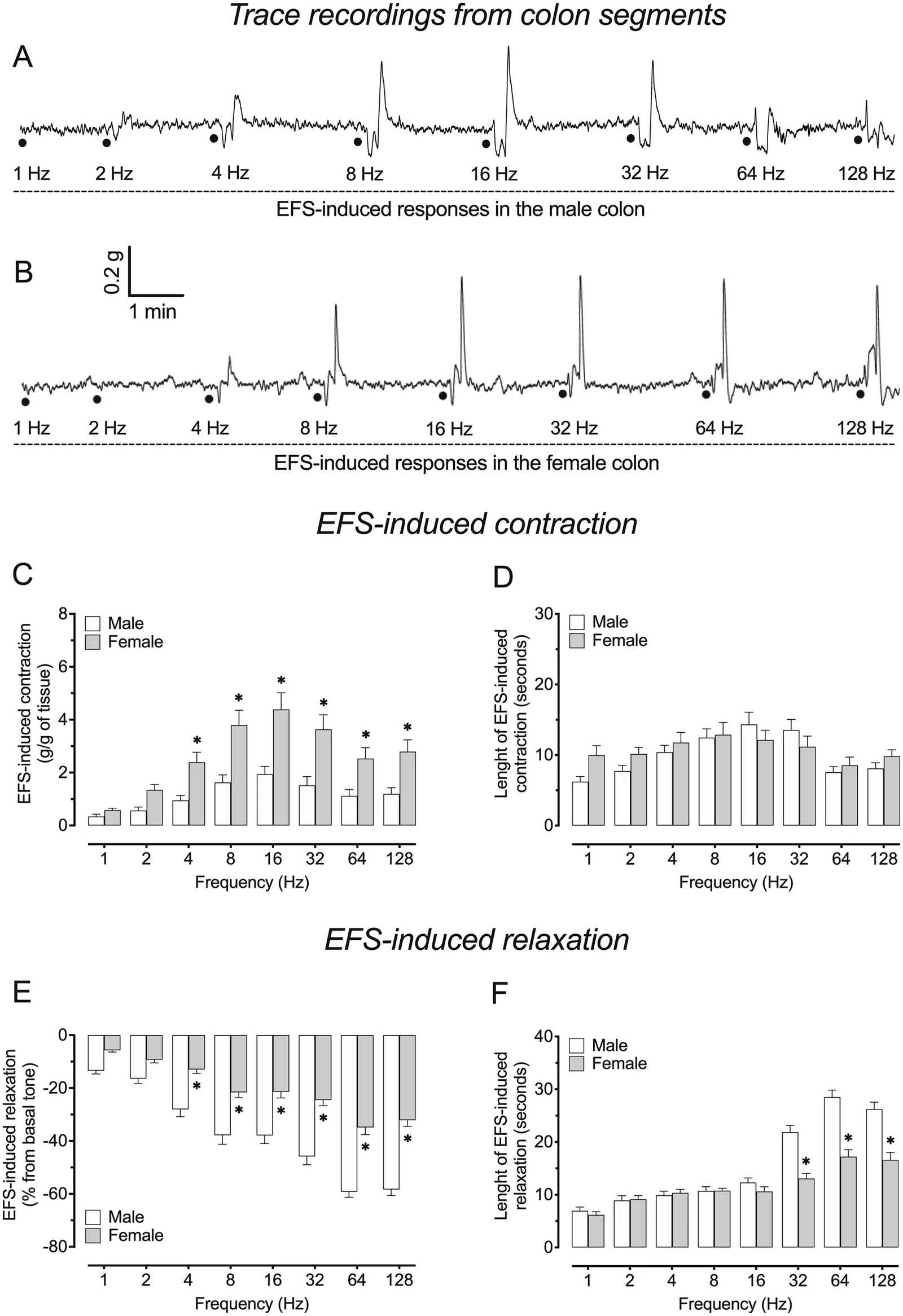
Contractile and relaxant responses evoked by electrical field stimulation (EFS) in colonic segments from male and female mice. Representative trace recordings illustrating frequency-response curves obtained in colonic preparations of male (**A**) and female (**B**) mice. The peak amplitude and duration of EFS-induced contractile (**C**, **D**) and relaxant (**E**, **F**) responses were quantified for each stimulation frequency. Results are presented as the mean ± standard error of the mean and were obtained from colonic segments of 30 animals per group. * indicates *P* < 0.05, difference between males and females for the same EFS frequency; two-way ANOVA followed by Šidák post hoc test

These experiments were performed using only colon segments, and EFS-evoked responses were measured as illustrated in Supplementary Fig. S1. The representative recordings shown in Fig. 7A and B demonstrate that, upon EFS, colonic preparations from male and female mice exhibited frequency-dependent biphasic responses, characterized by an initial relaxation followed by a contraction. Interestingly, the peak (Fig. 7C), but not the duration (Fig. 7D), of EFS-induced contraction was significantly greater in the colon of female mice. Furthermore, both the peak (Fig. 7E) and the duration (Fig. 7F) of EFS-evoked relaxation were shorter in female than in male colonic segments.

### Effects of acetylcholinesterase inhibition on colonic reactivity to EFS

**Fig. 8:**
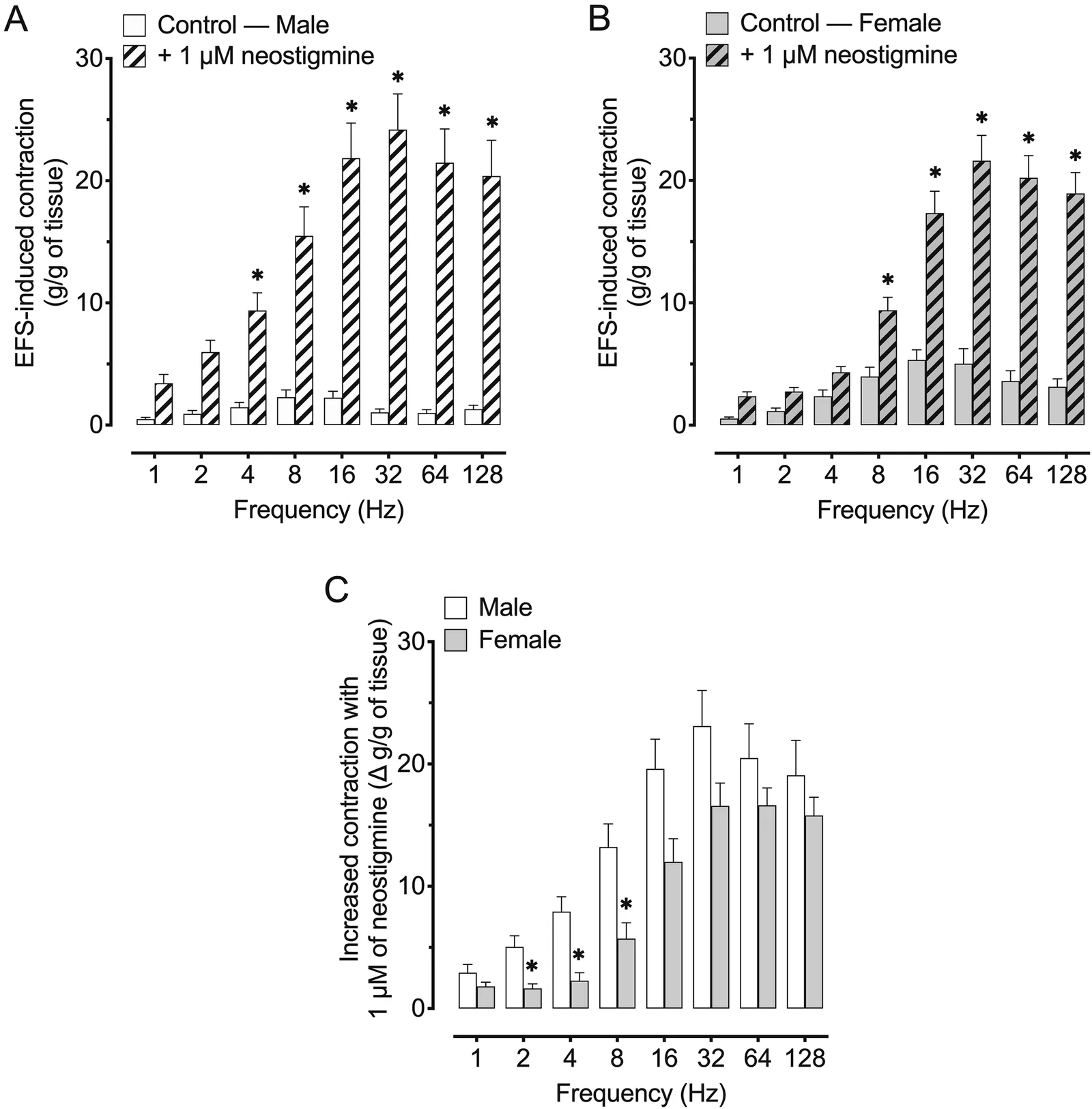
Effect of neostigmine on electrical field stimulation (EFS)-induced contractions in the colon of male and female mice. Incubation with 1 µM neostigmine enhanced contractile responses evoked by EFS in colonic preparations from male (**A**) and female (**B**) mice. The magnitude of the neostigmine-induced potentiation of EFS-evoked contractions is shown as the delta values (**C**). Results are presented as the mean ± standard error of the mean and were obtained from colonic segments of 12 animals per group. * indicates *P* < 0.05 between control and neostigmine groups (Panels A and B) or male and female groups (Panel C); two-way ANOVA followed by Šidák post hoc test

In this set of experiments, the effects of EFS were evaluated before and after incubation with the acetylcholinesterase inhibitor neostigmine, following the schematic drawing presented in the Supplementary Fig. S4A. Neostigmine induced a sustained contractile effect (Supplementary Fig. S4B), abolished EFS induced relaxation (data not shown), and potentiated EFS-induced contraction in colonic segments from both male (Fig. 8A) or female (Fig. 8B) mice. Notably, the magnitude of neostigmine-induced potentiation of EFS-evoked contraction was significantly greater in the colon preparations from male mice (Fig. 8C), thereby eliminating the sex-related differences found in responses to EFS before neostigmine incubation.

### Effects of nitric oxide synthase inhibition on colonic reactivity to EFS

**Fig. 9:**
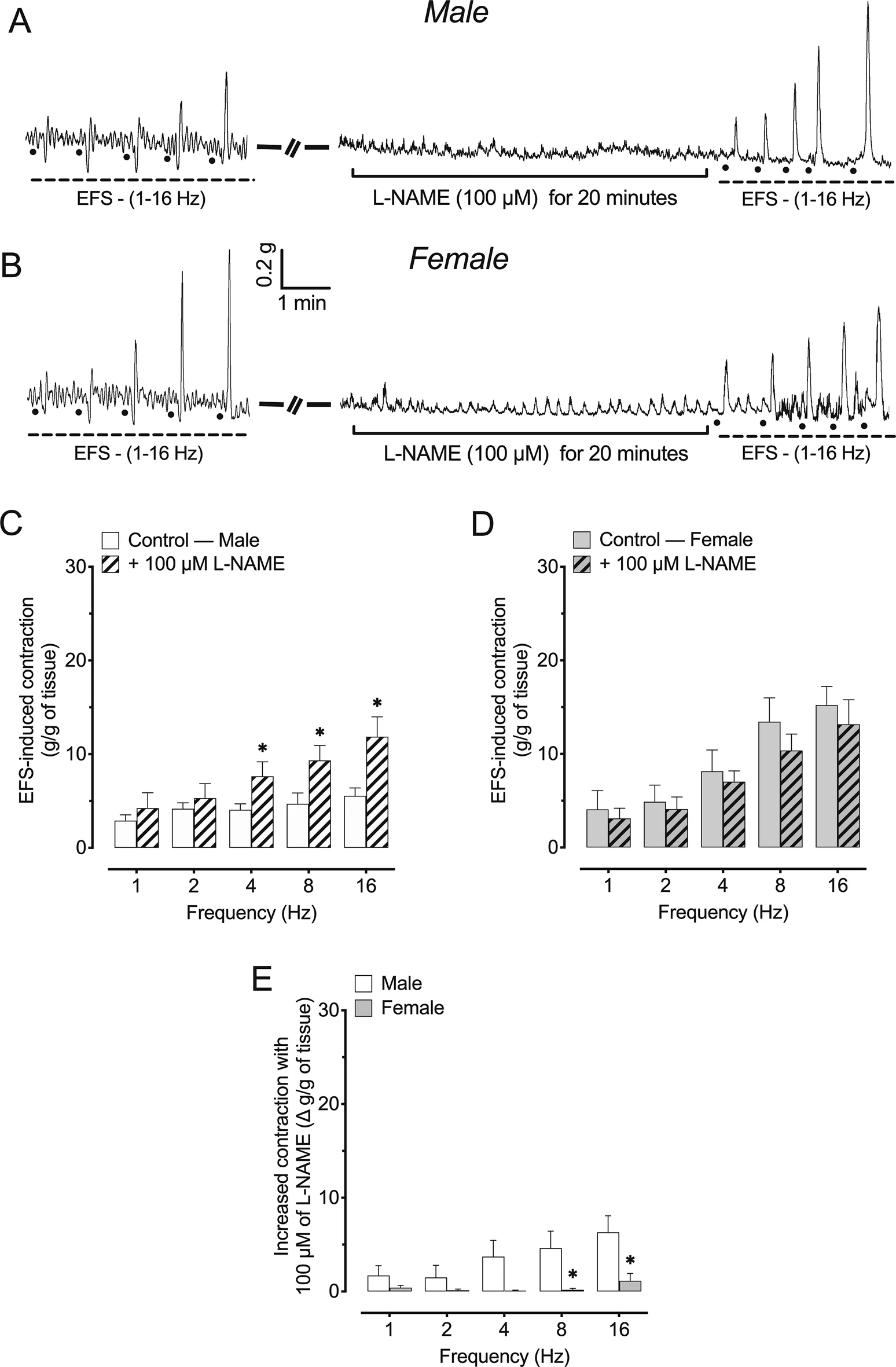
Effect of L-NAME on electrical field stimulation (EFS)-induced contractions in the colon of male and female mice. Representative trace recordings illustrating the effect of L-NAME on the frequency-response curves obtained in colonic preparations of male (**A**) and female (**B**) mice. Incubation with 100 µM L-NAME augmented the contractile responses evoked by EFS in colonic preparations from male (**C**) but not from female (**D**) mice. The magnitude of the neostigmine-induced potentiation of EFS-evoked contractions is shown as the delta values (**E**). Results are presented as the mean ± standard error of the mean and were obtained from colonic segments of 6 animals per group. * indicates *P* < 0.05 between control and L-NAME groups (Panel C) or male and female groups (Panel E); two-way ANOVA followed by Šidák post hoc test

Trace recordings showing the frequency-response curve generated by EFS-induced responses at increasing stimulation frequencies before and after incubation with L-NAME in colonic segments from male and female mice are shown in Fig. 9A and B, respectively. L-NAME nearly abolished the EFS-induced relaxation in the colon of both male and female mice (Supplementary Fig. S5A and B, respectively). Additionally, in colonic segments from male mice, the contractile responses evoked by EFS at 4–16 Hz were increased by approximately 80–100% in the presence of L-NAME (Fig. 9C). However, this potentiation was not found in colonic preparations from female mice (Fig. 9D). A direct comparison of the potentiating effect of L-NAME on EFS-evoked contractions in colonic preparations from male and female mice is shown in Fig. 9E.

## Discussion

The primary aim of this study was to investigate the existence of sex-related differences in gastrointestinal smooth muscle functionality. For this purpose, we employed the classical organ bath apparatus, widely regarded as the gold standard for in vitro functional studies. Although organ bath preparations have been used in physiology since the late 19th century, with intestinal tissues among the earliest experimental models, relatively few studies have included gastrointestinal segments from both male and female animals. To our knowledge, this is the first study to systematically characterize and compare the contractile and relaxant properties of isolated stomach, small intestine, and colonic preparations from male and female rodents. Although we observed only minor sex-related differences in upper gastrointestinal tract preparations, the colon of female mice showed markedly greater responsiveness to cholinergic stimulation.

Motility and contractility are two of the main pillars of gastrointestinal function and overall gut health. The coordinated interaction between these two components is essential for efficient digestion and gastrointestinal transit. Using organ baths, we evaluated the rate of spontaneous movements in all major segments of the gastrointestinal tract. Analyzing the amplitude of these movements also allowed us to compare the force generated by each preparation. In the absence of exogenous stimuli, both the gastric corpus and colonic preparations displayed the lowest frequency and force of the spontaneous contractions. This finding is consistent with the well-established weak basal pacemaker activity of the stomach in both rodents (Ordog et al. 1999) and humans (Sinn et al. 2010), as well as with the lower basal tone and low-frequency spontaneous contractility activity described in the mouse colon (Beck et al. 2018). Importantly, although preparations from male and female mice exhibit strictly similar patterns of spontaneous motility across the segments examined, the force generated by the ileum of females was significantly greater. Because the subsequent steps of our study focused on evoked responses, we did not further explore this finding, so we cannot propose mechanistic explanations at this stage. Nevertheless, sex-related differences in the regulation, activity, distribution, or density of interstitial cells of Cajal, which are electrically coupled to smooth muscle and act as pacemakers and modulators of gastrointestinal motility, are a reasonable hypothesis that warrants further investigation.

In addition to the spontaneous movements, we also evaluated the reactivity of the gastrointestinal segments to exogenous stimuli. Acetylcholine is the primary endogenous excitatory neurotransmitter mediating gastrointestinal smooth-muscle contraction. In contrast, under physiological autonomic regulation, norepinephrine generally opposes cholinergic excitation by inhibiting gastrointestinal contractions (De Ponti et al. 1996). Moreover, dopamine, another catecholamine produced and released by several cell types within the gastrointestinal tract, acts as a neuromodulator by decreasing acetylcholine release and, in some cases, directly affecting smooth muscle cells (for a review see Serio and Zizzo 2023). Only moderate sex-related differences were found in adrenergic responses in the gastric corpus and jejunum, whereas a markedly enhanced ACh-induced contractile response was observed in the colon of female mice, highlighting the potential importance of cholinergic mechanisms in sex-related differences in gastrointestinal function.

In smooth muscle cells, acetylcholine-mediated contractions occur primarily through activation of M2 and M3 muscarinic receptors (Unno et al. 2005), which are G-protein-coupled receptors widely expressed throughout the gastrointestinal system. The M3 receptor subtype signals via the phospholipase C-phosphatidylinositol pathway, leading to inositol trisphosphate-mediated calcium release from the sarcoplasmic reticulum and membrane depolarization, thereby elevating intracellular free calcium levels (Prestwich and Bolton 1995). Additionally, M3-receptor activation in the gut also stimulates the RhoA-Rho-kinase calcium-sensitization pathway (Perrino 2016). Consequently, the phasic and tonic components of cholinergic contraction arise from a combination of receptor-mediated intracellular calcium mobilization, extracellular calcium entry, Ca²⁺-dependent activation of the contractile machinery, and calcium-sensitization mechanisms. All these processes are counteracted by pathways activated by adrenergic and dopaminergic receptors. Thus, sex-related differences that reduce the activation or effectiveness of these relaxing pathways could mechanistically explain the enhanced colonic responses to acetylcholine in female mice. However, our data do not support this hypothesis, as no sex-related differences in the efficacy or potency of dopamine, norepinephrine, or isoprenaline were found in the colon.

An important finding came from experiments using carbamylcholine, another cholinergic agonist that, like acetylcholine, activates muscarinic and nicotinic receptors (Johnson et al. 2020). The absence of differences in colonic contractile responses to carbamylcholine suggests that alterations in muscarinic receptor function, downstream contractile signaling, or nicotinic receptor-mediated enteric neural activation are unlikely to account for the enhanced acetylcholine-induced contraction in colonic preparations from female mice. Unlike acetylcholine, however, carbamylcholine is resistant to hydrolysis by acetylcholinesterase, suggesting that differential acetylcholine degradation and consequent tissue availability may instead contribute to this sex-related difference.

One limitation of organ bath studies is the requirement for relatively high concentrations of exogenously applied drugs. Because we observed enhanced acetylcholine-induced contractile effects only at higher concentrations of this muscarinic agonist, we also subjected colonic segments to EFS, an approach in which the net contractile response depends solely on endogenously released excitatory and inhibitory neurotransmitters. EFS-evoked contractions in colonic segments are mediated mainly by acetylcholine release, as demonstrated by their sensitivity to atropine (Hasler et al. 1990). Our findings indicate that neural stimulation not only elicited augmented contractile responses but also reduced relaxation in colonic segments from female mice, further reinforcing the existence and biological importance of sex-related differences in the balance between contractile and relaxant mechanisms in the colon.

We next used a functional approach to investigate the role of acetylcholinesterase, a key regulator of cholinergic signaling that rapidly hydrolyses acetylcholine. To this end, we evaluated the EFS-evoked contractions in the presence of neostigmine, a well-known acetylcholinesterase inhibitor. Studies using similar approaches previously demonstrated that both the potency and efficacy of acetylcholinesterase-sensitive muscarinic receptor agonists depend on the acetylcholinesterase activity, which varies along different regions of the gastrointestinal tube (Chanda et al. 2010; Jarvie et al. 2008). Exposure to neostigmine alone produced a limited but sustained contraction of colonic segments in male and female mice, likely due to reduced degradation of spontaneously released acetylcholine within enteric cholinergic pathways. Although this initial response did not differ between preparations from male and female mice, the potentiation of EFS-evoked contractions produced by neostigmine was much higher in the colon from male mice, mainly at lower frequencies. Taking into account the reduced ability of neostigmine to potentiate EFS-induced contractions in colonic segments from female mice, together with the absence of sex-related differences in the contractile effects of carbamylcholine, our findings suggest that reduced acetylcholinesterase activity in the female colon may contribute to the enhanced contractile responses observed following both exogenous acetylcholine application and neural stimulation. Interestingly, reduced acetylcholine degradation may also contribute to the augmented contractile responses to KCl observed in the female colon. KCl-induced depolarization has previously been shown to activate voltage-gated calcium channels in the myenteric neurons, promoting the release of excitatory mediators, including acetylcholine (Shinozuka et al. 1985).

Given the wide range of inhibitory and excitatory neurotransmitters and neuromodulators involved in enteric neural circuits (for review see Sanders et al. 2012), multiple mediators may contribute to the differences observed in both acetylcholine- and EFS-mediated responses in the colon of male and female mice. Although this study did not comprehensively investigate the non-adrenergic and non-cholinergic mediators that may contribute to the sex-related differences described here, we examined the potential contribution of nitrergic neurotransmission, a major inhibitory pathway in the enteric nervous system. Previous studies have shown that inhibiting nitric oxide synthesis can reduce EFS-induced relaxation and potentiate the contractile component of EFS-induced responses in the colon of mice and humans (i.e., Ghia et al. 2004; Park et al. 2015). We found that the contribution of the nitrergic signaling to restraining the contractile component of EFS-induced responses appears to be smaller in the colon of females, since the non-selective nitric oxide synthase inhibitor L-NAME potentiated EFS-induced contractions in males but not in those from females. These results were particularly surprising because previous studies have provided evidence of enhanced nitrergic signaling in the colon of female rodents, both under basal conditions and following estrogen exposure (Nyavor et al. 2019; Shah et al. 2001). However, none of these studies directly examined the role of nitrergic signaling in colonic contractile function.

It is important to note that according to the data obtained using L-NAME, nitrergic transmission capable of producing relaxation is present in the colon of both male and female mice, suggesting that the mechanisms by which the nitrergic pathway mediates relaxation and restrains the contractile component of EFS-induced responses may be functionally dissociable and differentially regulated between sexes. Our data do not allow us to determine the mechanisms underlying this differential regulation. Nevertheless, because cholinergic and nitrergic pathways interact within enteric circuits, and our findings are consistent with greater acetylcholine responsiveness in the colon from females, sex-dependent differences in the balance between excitatory cholinergic and inhibitory nitrergic signaling may represent an important determinant of gastrointestinal motor function, both under physiological conditions and in disorders characterized by altered motility.

Recent advances in gastrointestinal research have highlighted the importance of the gut in maintaining homeostasis across multiple organ systems. Altogether, these findings have also underscored the importance of gastrointestinal motility as a key determinant of gut health. Consistent with this perspective, the present study provides evidence of sexual dimorphism in both acetylcholinesterase-dependent regulation of cholinergic signaling and nitrergic modulation of excitatory colonic smooth muscle responses in mice. If these findings were translatable to humans, they may provide important insights into the pathophysiology of several motility-related disorders that are more prevalent in women.

## Supplementary Information

Below is the link to the electronic supplementary material.

Supplementary Material 1

## Data Availability

All data are available from the authors upon reasonable request.
