# Supplementary material for: Sex-dependent regulation of cholinergic responses in the mouse colon: functional evidence for reduced acetylcholinesterase-dependent control and nitrergic modulation in females
Source: J Muscle Res Cell Motil. 2026 Sep 18;47(4):21. doi: 10.1007/s10974-026-09739-w (PMC13588863; doi:10.1007/s10974-026-09739-w)
Supplement: Supplementary file 1 [file 10974_2026_9739_MOESM1_ESM.pdf]

Supplementary Results

Sex-dependent regulation of cholinergic responses in the mouse colon: functional evidence for reduced acetylcholinesterase-dependent control and nitrergic modulation in females

Stherfanni Almeida Lima-Moura, José Eduardo da Silva-Santos

Laboratory of Cardiovascular and Smooth Muscle Biology, Department of Pharmacology, Center of Biological Sciences, Universidade Federal de Santa Catarina, Florianópolis, Santa Catarina, Brazil.

Research paper for: *Journal of Muscle Research and Cell Motility*

*Corresponding author: Laboratory of Cardiovascular and Smooth Muscle Biology, Department of Pharmacology, Universidade Federal de Santa Catarina – Campus Universitário Trindade – Center of Biological Sciences, Block D, Room 116. Florianópolis – SC, 88.040-900, Brazil.
Tel: +55 (48) 3721-4847

E-mail address: j.e.silva.santos@ufsc.br

Supplementary Figures

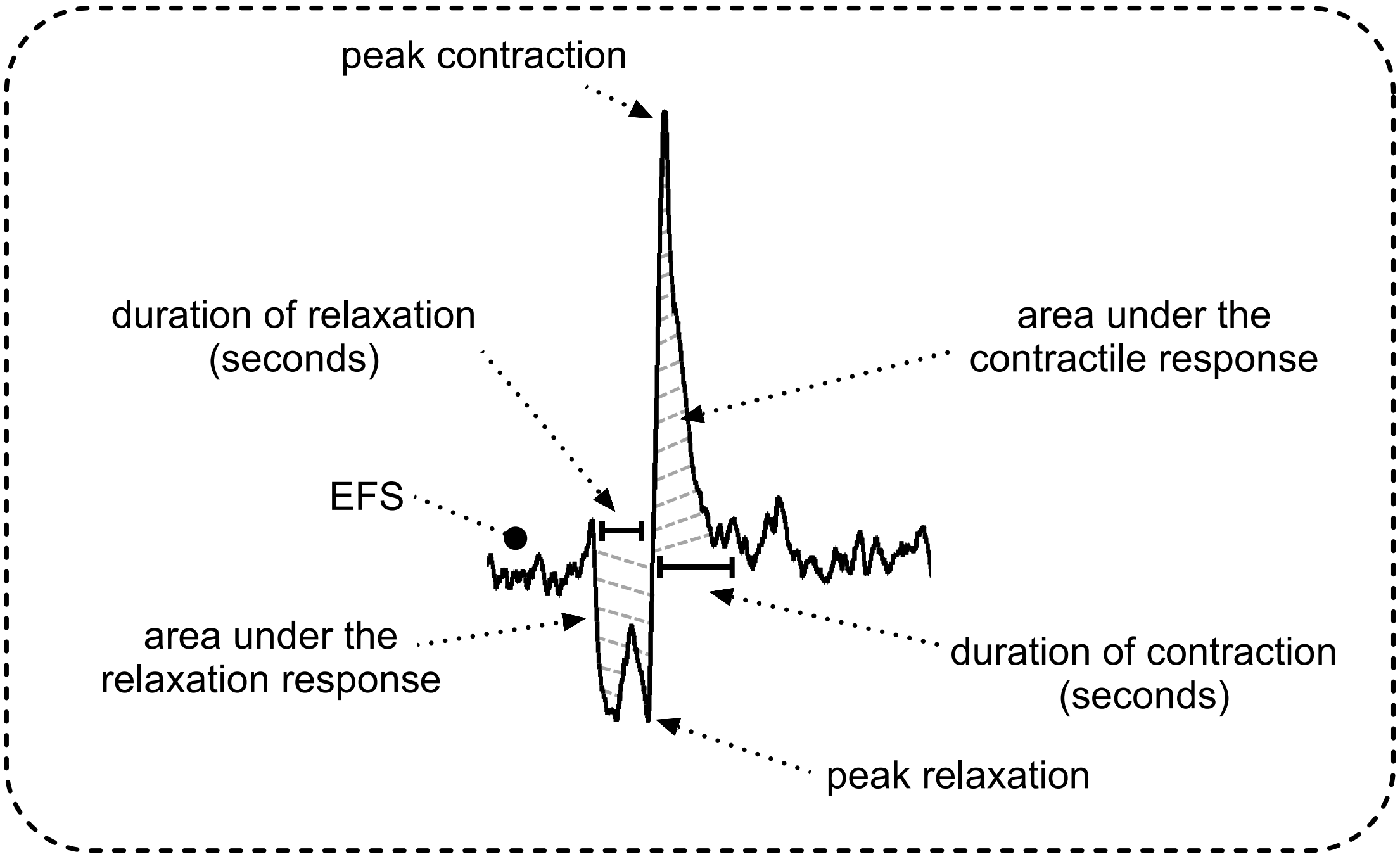

SUPPLEMENTARY FIG. S1 Illustration showing the parameters quantified to assess colonic responses to electrical field stimulation (EFS). A representative trace recording obtained from a colonic preparation subjected to EFS is shown. The round-head arrow indicates the time point at which EFS was applied to the preparation. Additional arrows indicate the regions of the trace analyzed to determine the peak amplitude, duration, and area under the curve of both the relaxant and contractile EFS-evoked responses.

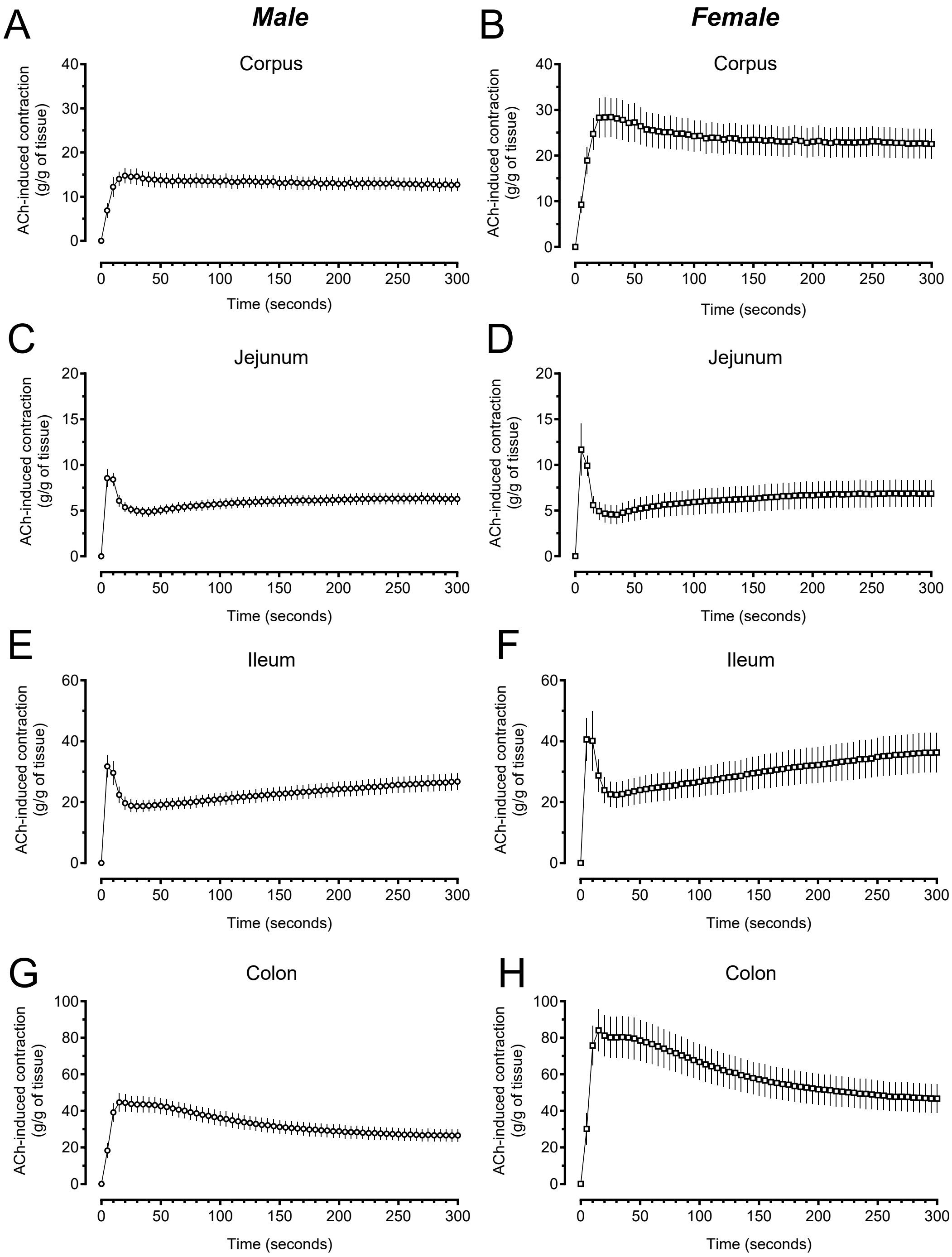

SUPPLEMENTARY FIG. S2 Contractile responses of gastrointestinal segments following the first exposure to acetylcholine (ACh). Time-course profiles of contractile responses recorded in gastric corpus (**A**, **B**), jejunum (**C**, **D**), ileum (**E**, **F**), and colon (**G**, **I**) of male (left panels) and female (right panels) mice following the first exposure to 30 μM ACh. Results are presented as mean ± standard error of the mean and were obtained from gastrointestinal segments of 12 animals per group.

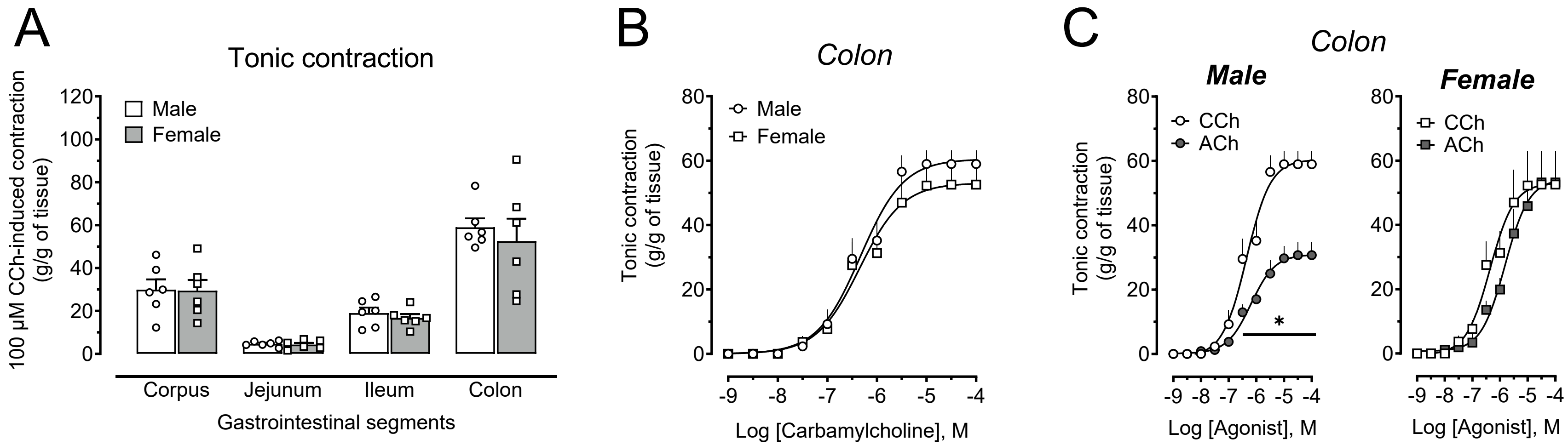

SUPPLEMENTARY FIG. S3 Tonic component of the contractile effect induced by carbamylcholine in the gastrointestinal segments of mice. Maximal contractile responses to carbamylcholine (CCh) in gastric corpus, jejunum, ileum, and colon preparations from male and female mice (**A**), and noncumulative concentration-response curves obtained in colonic preparations (**B**). For direct comparison, contractile responses evoked by noncumulative concentrations of CCh and acetylcholine (ACh) in the colon (**C**) from male and female mice are plotted together. All data included in this figure refer to the tonic component of the contractile responses and were measured in the same preparations used in Figure 5 of this study. Intestinal segments from six animals were used per group. * indicates $P < 0.05$, comparing the contractile responses elicited by CCh and ACh; two-way ANOVA followed by Šidák's post hoc test.

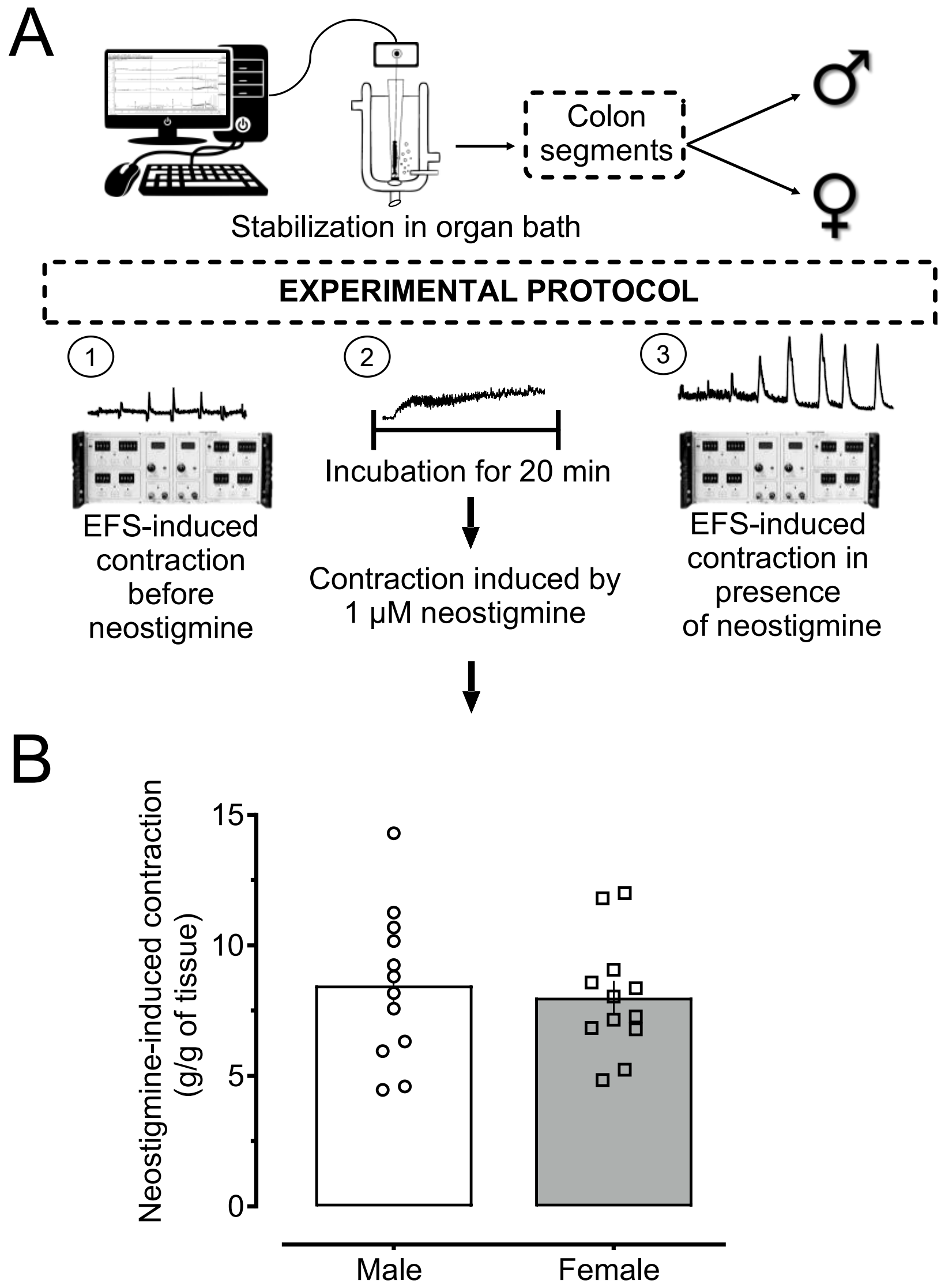

SUPPLEMENTARY FIG. S4 Schematic representation of the protocol using neostigmine. After mounting in the organ baths, colonic segments were allowed to stabilize (**A**) and were subjected to frequency-response curves before (1) and after (3) incubation with 1 μM neostigmine. The sustained contraction induced by neostigmine during the incubation period (2) was quantified and compared between colonic segments obtained from male and female mice (**B**). Results are presented as the mean ± standard error of the mean from colonic segments obtained from 12 animals per group.

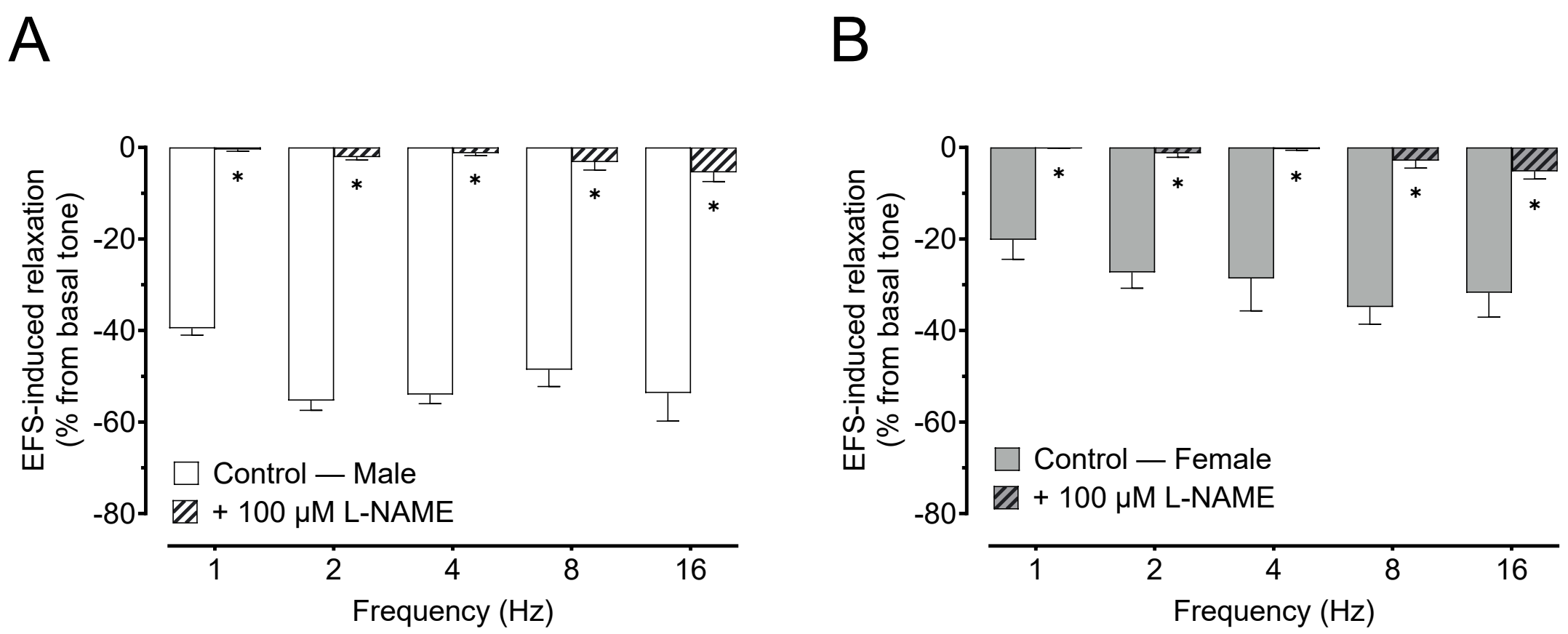

SUPPLEMENTARY FIG. S5 Relaxant responses evoked by electrical field stimulation (EFS) before and after incubation of L-NAME in colonic segments from male and female mice. Incubation with 100 µM L-NAME inhibited relaxation responses evoked by EFS in colonic preparations from male (**A**) and female (**B**) mice. Results are presented as the mean ± standard error of the mean from colonic segments obtained from six animals per group. * indicates $P < 0.05$ between control and L-NAME groups; two-way ANOVA followed by Šidák post hoc test.

Supplementary Tables

SUPPLEMENTARY TABLE S1: Spontaneous movements of gastrointestinal preparations from male and female mice.

		**Corpus**	**Jejunum**	**Ileum**	**Colon**
Frequency of the spontaneous movements (waves/min)	*Male*	$7.2 \pm 0.4^{*jj, il, cl}$	$46.9 \pm 1.5^{*cp, il, cl}$	$39.3 \pm 1.1^{*cp, jj, cl}$	$12.0 \pm 0.5^{*cp, jj, il}$
	Female	$7.5 \pm 0.3^{*jj, il}$	$47.8 \pm 1.9^{*cp, il, cl}$	$38.7 \pm 2.1^{*cp, jj, cl}$	$11.1 \pm 0.5^{*jj, il}$
Amplitude of the spontaneous movements (g/g)	*Male*	4.32 ± 1.04	$3.31 \pm 0.48^{*il}$	$6.65 \pm 0.91^{*jj, cl}$	$1.19 \pm 0.21^{*il}$
	Female	$5.34 \pm 0.96^{*il, cl}$	$4.46 \pm 0.63^{*il}$	$10.60 \pm 1.57^{*cp, cl; \#}$	$2.01 \pm 0.25^{*cp, il}$

Values are expressed as mean ± standard error of the mean obtained from intestinal segments of mice studied in organ baths. The data shown correspond to the descriptive statistics of results presented in Figure 2. Statistical analyses were performed using two-way ANOVA followed by Sidak's multiple comparison test. * followed by "cp", "jj", "il" or "cl" indicates $P < 0.05$ compared with the corpus, jejunum, ileum, or colon, respectively, from the same sex for the parameter evaluated; # indicates $P < 0.05$ compared with the corresponding parameter measured in gastrointestinal segments from male mice.

SUPPLEMENTARY TABLE S2: KCl-induced contraction in gastrointestinal segments of male and female mice.

		Corpus	Jejunum	Ileum	Colon
Maximal contraction (g/g of tissue)	*Male*	38.37 ± 6.97^{*jj}	13.01 ± 0.73$^{*cp, il, cl}$	41.27 ± 2.12^{*jj}	46.22 ± 4.71^{*jj}
	Female	38.90 ± 3.48$^{*jj, cl}$	14.36 ± 0.78$^{*cp, il, cl}$	40.51 ± 2.22$^{*jj, cl}$	58.09 ± 5.63$^{*cp, jj, il; \#}$

Values are expressed as mean ± standard error of the mean of responses to a 120 mM KCl-modified nutritive solution, recorded in intestinal segments from mice studied in organ baths. The data presented correspond to the descriptive statistics of the results shown in Figure 3. Statistical analyses were performed using two-way ANOVA followed by Sidak's multiple comparison test. * followed by "cp", "jj", "il" or "cl" indicates *P* < 0.05 compared with the corpus, jejunum, ileum, or colon segments, respectively, from the same sex; # indicates *P* < 0.05 compared with the corresponding gastrointestinal segment from male mice.

SUPPLEMENTARY TABLE S3: Phasic contraction induced by noncumulative concentrations of acetylcholine in gastrointestinal segments of male and female mice.

[ACh], µM		Corpus	Jejunum	Ileum	Colon
0.01	*Male*	0.38 ± 0.27	0.54 ± 0.32	1.87 ± 1.08	0.51 ± 0.28
	Female	0.71 ± 0.37	0.54 ± 0.36	2.09 ± 1.02	0.52 ± 0.23
0.03	*Male*	0.47 ± 0.31	1.16 ± 0.34	9.08 ± 1.41	1.68 ± 0.59
	Female	1.61 ± 0.97	2.63 ± 0.69	10.58 ± 3.04	5.38 ± 1.40
0.1	*Male*	1.97 ± 0.62	4.15 ± 0.87	12.98 ± 1.83	7.96 ± 3.15
	Female	4.14 ± 1.80	5.46 ± 1.27	18.26 ± 1.94	11.78 ± 2.05
0.3	*Male*	6.22 ± 2.34	8.44 ± 1.64	24.59 ± 2.18	13.56 ± 4.92
	Female	11.02 ± 2.21	9.46 ± 2.08	28.83 ± 3.24	18.21 ± 1.71
1	*Male*	12.11 ± 2.91	11.00 ± 1.64	36.32 ± 3.06	25.83 ± 4.87
	Female	19.23 ± 3.47	13.49 ± 1.26	35.69 ± 2.67	29.89 ± 3.71
3	*Male*	19.30 ± 5.43	12.72 ± 1.89	41.63 ± 4.95	29.33 ± 4.51
	Female	23.50 ± 4.51	15.11 ± 1.92	46.27 ± 2.99	40.32 ± 1.45
10	*Male*	22.24 ± 5.51	14.38 ± 1.92	48.01 ± 6.52	38.01 ± 6.02
	Female	29.65 ± 4.71	16.57 ± 1.76	55.85 ± 2.47	56.11 ± 4.43*
30	*Male*	27.44 ± 7.06	15.24 ± 2.11	51.38 ± 7.14	44.20 ± 7.05
	Female	32.63 ± 4.88	18.03 ± 2.19	60.56 ±3.65	70.42 ± 4.63*
100	*Male*	34.19 ± 8.52	15.64 ± 1.97	53.12 ± 7.24	50.95 ± 7.24
	Female	37.49 ± 5.29	18.29 ± 2.09	60.69 ± 3.63	85.79 ± 6.33*
300	*Male*	35.66 ± 8.04	15.64 ± 1.97	53.12 ± 7.24	50.95 ± 7.24
	Female	41.12 ± 6.16	18.52 ± 2.22	62.04 ± 3.69	86.58 ± 6.05*

Values are expressed as mean ± standard error of the mean of phasic contraction (see Figure 1E for methodological details) induced by noncumulative concentrations of acetylcholine (ACh) in intestinal segments from mice. The data presented correspond to the descriptive statistics of the results shown in Figure 4. Statistical analyses were performed using two-way ANOVA followed by Sidak's multiple comparison test. * indicates $P < 0.05$ compared with the corresponding ACh concentration and gastrointestinal segment from male mice.

SUPPLEMENTARY TABLE S4: Tonic contraction induced by noncumulative concentrations of acetylcholine in gastrointestinal segments of male and female mice.

[ACh], µM		Corpus	Jejunum	Ileum	Colon
0.01	*Male*	0.00 ± 0.00	0.04 ± 0.03	0.07 ± 0.07	0.00 ± 0.00
	Female	0.14 ± 0.11	1.24 ± 1.24	1.91 ± 1.91	0.00 ± 0.00
0.03	*Male*	0.00 ± 0.00	0.40 ± 0.28	0.11 ± 0.11	0.86 ± 0.81
	Female	0.57 ± 0.26	2.10 ± 1.37	2.06 ± 2.06	1.18 ± 1.18
0.1	*Male*	0.64 ± 0.40	1.38 ± 0.62	1.48 ± 0.34	1.25 ± 1.07
	Female	1.83 ± 0.90	3.29 ± 12.05	3.98 ± 2.49	1.93 ± 1.58
0.3	*Male*	3.49 ± 1.92	3.19 ± 0.90	4.29 ± 0.78	3.76 ± 0.99
	Female	4.55 ± 1.52	4.66 ± 1.98	9.08 ± 4.20	3.38 ± 2.75
1	*Male*	9.95 ± 3.01	4.15 ± 1.02	14.95 ± 2.14	12.93 ± 2.52
	Female	14.73 ± 3.72	6.87 ± 1.96	17.73 ± 3.25	13.59 ± 2.79
3	*Male*	15.06 ± 5.43	4.99 ± 0.77	18.71 ± 2.69	17.02 ± 2.21
	Female	18.99 ± 4.08	7.38 ± 1.85	23.48 ± 4.19	19.91 ± 3.56
10	*Male*	20.63 ± 6.18	5.14 ± 0.67	21.11 ± 2.69	25.00 ± 4.07
	Female	27.21 ± 4.99	7.62 ± 1.78	25.77 ± 3.59	37.35 ± 7.72
30	*Male*	24.26 ± 8.27	5.14 ± 0.67	21.46 ± 2.64	29.73 ± 3.74
	Female	32.26 ± 5.90	7.62 ± 1.78	26.12 ± 3.52	45.86 ± 7.24*
100	*Male*	27.34 ± 8.63	5.14 ± 0.67	21.46 ± 2.64	30.71 ± 3.98
	Female	37.20 ± 6.69	7.62 ± 1.78	26.12 ± 3.52	53.20 ± 7.71*
300	*Male*	31.58 ± 10.49	5.14 ± 0.67	21.46 ± 2.64	30.71 ± 3.98
	Female	39.25 ± 7.21	7.62 ± 1.78	26.12 ± 3.52	53.23 ± 7.69*

Values are expressed as mean ± standard error of the mean of tonic contraction (see Figure 1E for methodological details) induced by noncumulative concentrations of acetylcholine (ACh) in intestinal segments from mice. The data presented correspond to the descriptive statistics of the results shown in Figure 4. Statistical analyses were performed using two-way ANOVA followed by Sidak's multiple comparison test. * indicates $P < 0.05$ compared with the corresponding ACh concentration and gastrointestinal segment from male mice.

SUPPLEMENTARY TABLE S5: Comparison of the maximal contractile effects (E_{max}) and half maximal effective concentration (EC_{50}) of acetylcholine and carbamylcholine (phasic contraction) in gastrointestinal segments from male and female mice.

**Phasic contraction/segment**		**Corpus**	**Jejunum**	**Ileum**	**Colon**
Emax (g/g of tissue)					
Male	Acetylcholine	$35.6 \pm 8^{*jj,il}$	$15.6 \pm 1.9^{*cp, il, cl}$	$53.1 \pm 7.2^{*jj, cp}$	$50.9 \pm 7.2^{*jj}$
	Carbamylcholine	$33.0 \pm 7.1^{*jj, il, cl}$	$14.5 \pm 1.1^{*cp, il, cl}$	$51.2 \pm 2.8^{*cp, jj, cl}$	$93.1 \pm 3.6^{*cp, jj, il; \#}$
Female	Acetylcholine	$41.1 \pm 6.1^{*jj, il, cl}$	$18.5 \pm 2.2^{*cp, il, cl}$	$62.0 \pm 3.7^{*cp, jj, cl}$	$85.8 \pm 6.3^{*cp, jj, il}$
	Carbamylcholine	$34.3 \pm 5.4^{*jj, il, cl}$	$16.8 \pm 1.7^{*cp, il, cl}$	$50.2 \pm 2.4^{*cp, jj, cl; \#}$	$88.5 \pm 8.4^{*cp, jj, il}$
EC_{50} (μM 95% confidence interval)					
Male	Acetylcholine	0.87 (0.2-4.0)	0.09 (0.04-0.24)	0.14 (0.06-0.34)	0.44 (0.15-1.36)
	Carbamylcholine	0.10 (0.02-0.46)	0.18 (0.11-0.30)	0.26 (0.18-0.39)	$0.52\ (0.35\text{-}0.78)^{*jj}$
Female	Acetylcholine	0.42 (0.15-1.2)	0.09 (0.04-0.23)	0.21 (0.11-0.36)	$1.36\ (0.83\text{-}2.21)^{*jj, il}$
	Carbamylcholine	0.07 (0.02-0.20)	0.38 (0.18-0.80)	0.32 (0.21-0.50)	0.42 (0.22-0.82)

Values are expressed as mean ± standard error of the mean of the maximal phasic contraction (Emax, in g/g) and the half-maximal effective concentration (EC_{50}, in μM, with 95% confidence interval) obtained from noncumulative concentration-response curves for each muscarinic agent. The data presented correspond to the descriptive statistics of the results shown in Figure 5. Statistical analyses were performed using two-way ANOVA followed by Sidak's multiple comparison test. * followed by "cp", "jj", "il" or "cl" indicates $P < 0.05$ compared with the corpus, jejunum, ileum, or colon segments, respectively, from the same sex; $^{\#}$ indicates $P < 0.05$ compared with acetylcholine-evoked responses in the same segment and sex.

SUPPLEMENTARY TABLE S6: Comparison of the maximal contractile effects (E_{max}) and half maximal effective concentration (EC_{50}) of acetylcholine and carbamylcholine (tonic contraction) in gastrointestinal segments from male and female mice.

Tonic contraction/segment		Corpus	Jejunum	Ileum	Colon
Emax (g/g of tissue)					
Male	Acetylcholine	31.5 ± 10.5*^{jj}	5.1 ± 0.6*$^{cp,\ il,\ cl}$	21.4 ± 2.6*^{jj}	30.7 ± 3.9*^{jj}
	Carbamylcholine	29.9 ± 4.8*$^{jj,\ il,\ cl}$	4.6 ± 0.5*$^{cp,\ il,\ cl}$	18.9 ± 2.5*$^{jj,\ cl}$	58.9 ± 4.1*$^{cp,\ jj,\ il;\ \#}$
Female	Acetylcholine	39.2 ± 7.2*^{jj}	7.6 ± 1.7*$^{cp,\ il,\ cl}$	26.1 ± 3.5*$^{jj,\ cl}$	53.2 ± 7.7*$^{jj,\ il}$
	Carbamylcholine	29.4 ± 5.0*$^{jj,\ cl}$	4.3 ± 0.8*$^{cp,\ cl}$	16.6 ± 1.8*^{cl}	52.5 ± 10.4*$^{cp,\ jj,\ il}$
EC_{50} (μM 95% confidence interval)					
Male	Acetylcholine	0.91 (0.16-5.42)	0.06 (0.02-0.17)	0.2 (0.11-0.36)	0.64 (0.32-1.28)*^{jj}
	Carbamylcholine	0.18 (0.08-0.44)	0.14 (0.07-0.28)	0.32 (0.17-0.62)	0.43 (0.25-0.73)
Female	Acetylcholine	0.87 (0.31-2.44)	0.06 (0.005-0.61)	0.18 (0.07-0.44)	1.54 (0.74-3.17)*$^{il,\ jj}$
	Carbamylcholine	0.10 (0.04-0.29)$^{\#}$	0.19 (0.08-0.46)	0.29 (0.17-0.50)	0.43 (0.15-1.25)

Values are expressed as mean ± standard error of the mean of the maximal tonic contraction (Emax, in g/g) and the half-maximal effective concentration (EC_{50}, in μM 95% confidence interval) obtained from noncumulative concentration-response curves for each muscarinic agent. The data presented correspond to the descriptive statistics of the results shown in Figure 5. Statistical analyses were performed using two-way ANOVA followed by Sidak's multiple comparison test. * followed by "cp", "jj", "il" or "cl" indicates $P < 0.05$ compared with the corpus, jejunum, ileum, or colon segments, respectively, from the same sex; $^{\#}$ indicates $P < 0.05$ compared with acetylcholine-evoked responses in the same segment and sex.

SUPPLEMENTARY TABLE S7: Relaxant responses of gastrointestinal segments from male and female mice to adrenergic and dopaminergic ligands.

		**Dopamine**		**Norepinephrine**		**Isoprenaline**	
		E_{max}	pD2	E_{max}	pD2	E_{max}	pD2
Corpus	*Male*	$57.3 \pm 9.9^{*jj, il, cl}$	5.04 ± 0.28	$104.4 \pm 1.8^{†}$	$7.02 \pm 0.05^{†}$	$97.2 \pm 4.6^{*jj, cl; †}$	$7.55 \pm 0.14^{†}$
	Female	$45.6 \pm 8.9^{*jj, il, cl}$	4.86 ± 0.18	$106.7 \pm 5.4^{*jj, cl; †}$	$6.91 \pm 0.16^{†}$	$116.8 \pm 3.1^{*jj; \#; †}$	$7.83 \pm 0.10^{†; \&}$
Jejunum	*Male*	$92.4 \pm 2.9^{*cp}$	4.72 ± 0.12	110.0 ± 6.0	$7.18 \pm 0.12^{†}$	$119.1 \pm 9.1^{*cp}$	$8.32 \pm 0.09^{†; \&}$
	Female	$104.9 \pm 9.9^{*cp}$	4.98 ± 0.15	$125.3 \pm 4.5^{*cp, il; \#}$	$7.11 \pm 0.15^{†}$	$143.0 \pm 10.9^{*il; \#; †}$	$8.52 \pm 0.13^{†; \&}$
Ileum	*Male*	$82.0 \pm 1.9^{*cp}$	4.90 ± 0.10	$100.1 \pm 3.3^{†}$	$7.28 \pm 0.18^{†}$	$102.6 \pm 3.2^{†}$	$8.47 \pm 0.14^{†; \&}$
	Female	$81.8 \pm 3.5^{*cp}$	4.86 ± 0.10	$98.3 \pm 3.1^{*jj, cl; †}$	$6.97 \pm 0.18^{†}$	$106.7 \pm 2.2^{*jj; †}$	$8.54 \pm 0.09^{†; \&}$
Colon	*Male*	$102.3 \pm 2.1^{*cp}$	5.18 ± 0.20	$115.2 \pm 2.9^{†}$	$7.56 \pm 0.09^{†}$	$118.8 \pm 4.4^{*cp; †}$	$8.73 \pm 0.10^{†; \&}$
	Female	$103.4 \pm 2.7^{*cp}$	5.09 ± 0.09	$124.1 \pm 3.0^{*cp, il; †}$	$7.16 \pm 0.23^{†}$	$128.2 \pm 5.4^{†}$	$8.89 \pm 0.09^{†; \&}$

Values show the mean ± standard error of the mean of the maximal relaxation (Emax, in %) and the negative logarithm of the half-maximal effective concentration (pD2), derived from cumulative concentration-response curves for each pharmacological agent. The data presented correspond to the descriptive statistics of the results shown in Figure 6. Statistical analyses were performed using two-way ANOVA followed by Sidak's multiple comparison test. * followed by "cp", "jj", "il" or "cl" indicates $P < 0.05$ compared with the corpus, jejunum, ileum, or colon segments, respectively, from the same sex; $^{\#}$ indicates $P < 0.05$ between male and female mice within the same gastrointestinal segment. † indicates $P < 0.05$ for Emax or pD2 between norepinephrine or isoprenaline and dopamine within the same segment and sex; $^{\&}$ indicates $P < 0.05$ for pD2, compared with norepinephrine.
